# A comparative UHPLC-Q/TOF–MS-based eco-metabolomics approach reveals temperature adaptation of four *Nepenthes* species

**DOI:** 10.1038/s41598-020-78873-3

**Published:** 2020-12-14

**Authors:** Changi Wong, Yee Soon Ling, Julia Lih Suan Wee, Aazani Mujahid, Moritz Müller

**Affiliations:** 1 Faculty of Engineering, Computing and Science, Swinburne University of Technology Sarawak, 93350 Kuching, Malaysia; 2 Water Research Unit, Faculty of Science and Natural Resources, University Malaysia Sabah, 88400 Kota Kinabalu, Sabah Malaysia; 3 Faculty of Resource Science and Technology, Universiti Malaysia Sarawak, 93400 Kota Samarahan, Sarawak Malaysia; 4 Institute of Biodiversity and Environmental Conservation, Universiti Malaysia Sarawak, 94300 Kota Samarahan, Sarawak Malaysia

**Keywords:** Metabolomics, Ecophysiology, Palaeoecology, Tropical ecology, Plant stress responses

## Abstract

*Nepenthes*, as the largest family of carnivorous plants, is found with an extensive geographical distribution throughout the Malay Archipelago, specifically in Borneo, Philippines, and Sumatra. Highland species are able to tolerate cold stress and lowland species heat stress. Our current understanding on the adaptation or survival mechanisms acquired by the different *Nepenthes* species to their climatic conditions at the phytochemical level is, however, limited. In this study, we applied an eco-metabolomics approach to identify temperature stressed individual metabolic fingerprints of four *Nepenthes* species: the lowlanders *N*. *ampullaria*, *N*. *rafflesiana* and *N*. *northiana*, and the highlander *N*. *minima*. We hypothesized that distinct metabolite regulation patterns exist between the *Nepenthes* species due to their adaptation towards different geographical and altitudinal distribution. Our results revealed not only distinct temperature stress induced metabolite fingerprints for each *Nepenthes* species, but also shared metabolic response and adaptation strategies. The interspecific responses and adaptation of *N*. *rafflesiana* and *N*. *northiana* likely reflected their natural habitat niches. Moreover, our study also indicates the potential of lowlanders, especially *N*. *ampullaria* and *N*. *rafflesiana*, to produce metabolites needed to deal with increased temperatures, offering hope for the plant genus and future adaption in times of changing climate.

## Introduction

*Nepenthes* (*N.*), the sole genus under the family *Nepenthaceae*, is one of the largest families of carnivorous plants, with an extensive geographical distribution across the Malay Archipelago, specifically in Borneo, Philippines, and Sumatra. To date, 151 species have been documented, with most species displaying high degrees of endemism and often restricted to single areas, i.e. *N. villosa*, *rajah* and *burbidgeae* which can only be found in Mount Kinabalu and the neighboring Mount Tambuyukon in Borneo^1–3^. The characteristic pitcher and their adaptation to nutrient poor soils has been well documented^4–7^.

*Nepenthes* can be clustered into two groups: lowlanders (with altitudinal distributions below 1100 m above sea level (asl)—hot and humid jungles) which can tolerate heat stress and highlanders (with altitudinal distributions beyond 1100 masl such as highland montane forests with warm days and cool to cold, humid nights) which can tolerate cold stress^2,8,9^. There are some exemptions such as *Nepenthes ampullaria* and *N*. *rafflesiana*, even though categorized as lowland species, both were recorded in highland environments but only very rarely^2–5^. Besides that, *N*. *minima* was the only highlander species able to grow well at our greenhouse under lowland conditions. Our current understanding on the adaptation or survival mechanisms acquired by the different *Nepenthes* species to their climatic conditions at the phytochemical level is, however, limited.

Heat stress has been shown to increase respiration, reduce photosynthesis, disrupt plant cellular structures and defensive mechanisms, and elevate stress metabolites production in plants^10–12^. Low temperature stress, on the other hand, can affect the photosynthesis rate of the plant thus causes the imbalance of the energy metabolism. Besides that, cellular DNA damage, physiological functions and metabolic sink disruption of plant cell were also recorded^13–16^. Both stresses will cause overproduction of reactive nitrogen species (RNS) and reactive oxygen species (ROS), thus causing oxidative stress in plants^17–20^.

Metabolomics can contribute significantly to our understanding of stress responses in plants by identifying the involved metabolites in response to endogenous and exogenous stressor^21–24^. Applying a nontargeted metabolome approach, combined with high-resolution Mass Spectrometry (MS) and high-resolution chromatography, we can discover true dynamics of biological systems in response to specific perturbations^25^.

In the current study, we investigated the impact of heat and cold stress on four *Nepenthes* species, representing lowland (*N*. *ampullaria*, *N*. *rafflesiana* and *N*. *northiana*) and highland species (*N*. *minima*). Using a MS-based non-targeted approach, we aim to understand how the underlying ecological adaptation of the plant species influences their metabolite regulation upon heat and cold exposure. Do they share (a) similar response toward the provided environmental conditions; or (b) are different strategies applied by each species?

## Results

**Figure 1:**
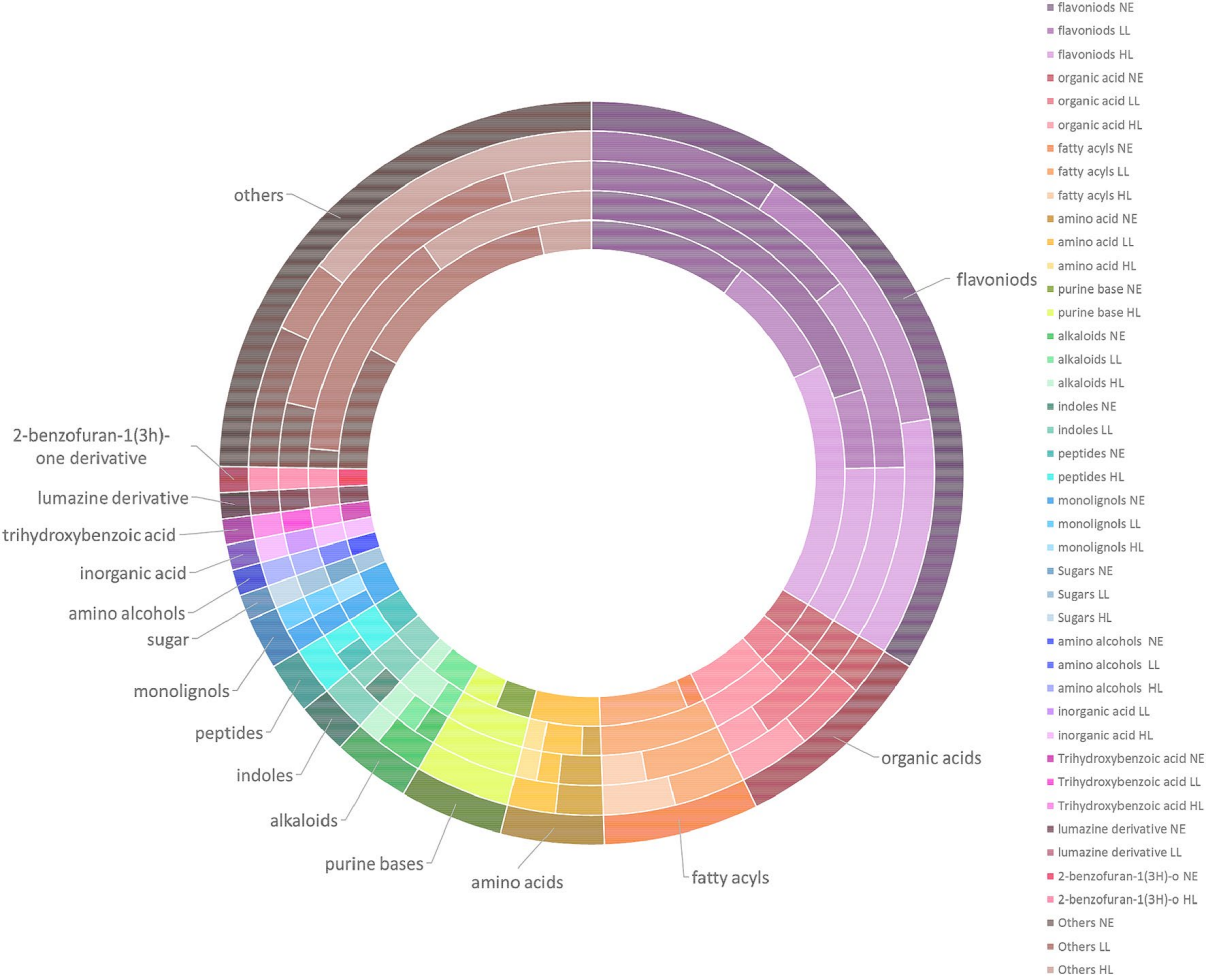
Pie chart depicting percentages of the 89 putatively identified metabolites from each of the metabolite groups, as well as the regulation of the metabolites of each group in response to the different temperature conditions. Each of the layer indicate a *Nepenthes* species, from inner layer to outer layer: *N.* *minima*, *ampullaria*, *northiana*, *rafflesiana*. *NE* no effect/particular pattern, *LL* metabolites expressed highest at lowland condition, *HL* metabolites expressed highest at highland condition.

We determined the individual metabolite fingerprints of four (4) *Nepenthes* species in response to highland, intermediate and lowland growing conditions. The applied workflow allowed the determination of 125 significantly altered metabolites (see Supplementary Table S1) under the provided conditions from which 89 could be identified. The identified metabolites were grouped under 16 categories, with the majority of them being flavonoids, followed by organic acids, fatty acyls, amino acids, purine base, alkaloids and some others (Fig. 1). Fourteen of the identified metabolites were found to be involved in 32 metabolic pathways (Table 1).

**Table 1:** Summary of highly correlated metabolites in different biosynthetic pathways.

Biosynthetic pathways	p-value	Metabolites
Phenylpropanoid derivatives biosynthesis^a^	2.56e−05	Quercetin^a,b,c,d,e,f,g,j,q^
Superpathway of flavones and derivatives biosynthesis^b^	4.65e−04	Luteolin^a,d,g,j,n^
Flavonols biosynthesis^c^	7.48e−04	Syringin^a,j,k^
Flavonoids biosynthesis^d^	1.18e−03	Kaempferol-3-glucoside^a,b,c,d,j^
Rutin biosynthesis^e^	2.95e−03	Rutin^a,b,c,d,e,j^
Quercetin glycoside biosynthesis (Arabidopsis)^f^	5.07E−03	Benzoate^a,j,m,p^
Flavonoid biosynthesis (In Equisetum)^g^	7.72e−03	Coniferin^a,j,k,p,r^
Proteinogenic amino acids biosynthesis^h^	2.26e−03	Quercetin 3-*O*-rhamnoside^a,b,c,d,f,j^
Amino acids biosynthesis^h^	6.54e−03	l-Arginine^h,i,p,s^
Proteinogenic amino acids degradation^i^	8.72e−03	l-Tryptophan^h,i,j,p^
Amino acids degradation^i^	1.07e−02	Adenine^h,l,p,t^
Secondary metabolites biosynthesis^j^	1.29e−02	l-Isoleucine^h,i^
Lignins biosynthesis^k^	2.31e−02	Adenosine^h,i,o,p,t^
S-methyl-5′-thioadenosine degradation ii^l^	2.59E−02	l-Isoleucine^p^
Benzoyl-β-d-glucopyranose biosynthesis^m^	2.59E−02	
Benzoate degradation^m^	3.86e−02	
Benzoate degradation II (aerobic and anaerobic)^m^	3.86E−02	
Chrysoeriol biosynthesis^n^	3.23e−02	
Adenine and adenosine salvage VI^o^	3.23E−02	
Degradation/utilization/assimilation^p^	3.35e−02	
Methylquercetin biosynthesis^q^	3.86e−02	
Phenylpropanoid derivatives degradation^r^	4.49e−02	
Coniferin metabolism^r^	4.49e−02	
Putrescine biosynthesis I^s^	4.49E−02	
Adenine and adenosine salvage II^t^	8.13E−04	
Adenine and adenosine salvage^t^	1.72E−03	
Purine Nucleosides Salvage II (Plant)^t^	2.95E−03	
Cytokinins degradation^t^	3.43e−03	
Purine nucleotide salvage^t^	7.72e−03	
l-methionine salvage^t^	2.19E−02	
l-methionine biosynthesis^t^	3.06E−02	
Purine nucleotide biosynthesis^t^	4.04e−02	

Letter(s) after the pathways and metabolites indicate the involved metabolites in the same pathway. Enrichment analysis was carried out using the Fisher Exact statistical test.

### Individual metabolomic fingerprints of the four *Nepenthes* species

**Figure 2:**
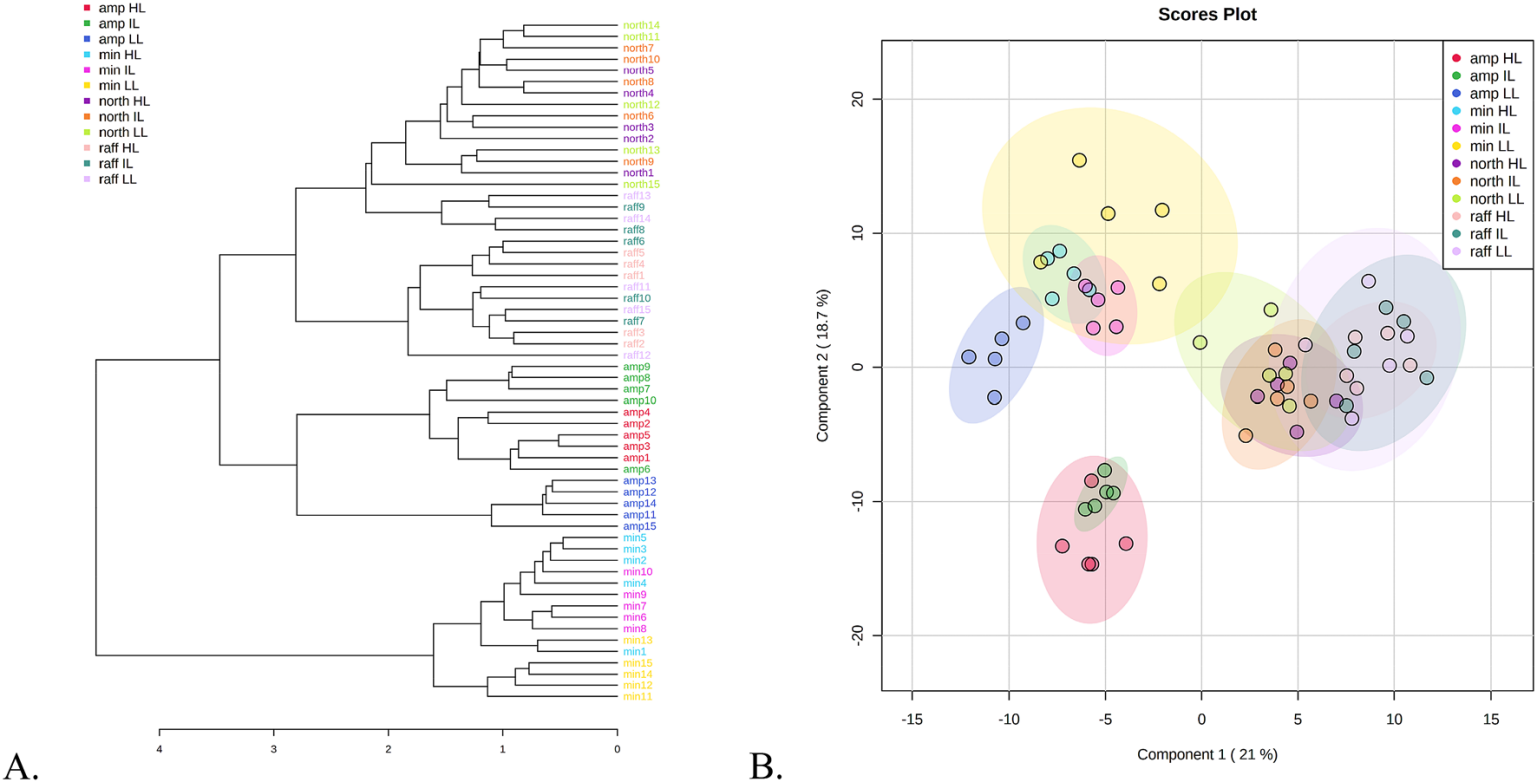
Metabolites profiles of the four *Nepenthes* species as influenced by the three different growing conditions. (**A**) Dendrogram showing the relationship among the samples using Pearson distances and average clustering. (**B**) Partial least squares–discriminant analysis (PLS-DA) score plot showing the spatial separation between the *Nepenthes* species and the provided growing conditions. Abbreviations: amp represents *N*. *ampullaria*, min represents *N*. *minima*, north represent *N*. *northiana*, and raff represents *N*. *rafflesiana*. The numbering after the species name represent the biological replicates. *HL* highland condition, *IL* intermediate condition, *LL* lowland condition.

The metabolomes of the four *Nepenthes* species, subjected to varying temperature regimes, displayed significant differences (p < 0.01; Supplementary Table S2). A dendrogram based on pearson distances and average clustering showed very distinct grouping of *N. ampullaria* and *N*. *minima* under all three temperature conditions compared to *N*. *northiana* and *N*. *rafflesiana* which were a little bit more mixed (Fig. 2A). Principle components 1 and 2 derived from the PLS-DA, showed the total variance among the species at 39.7% (Fig. 2B). Despite grouping distinctly on its own, PLS-DA indicated greater temperature-related variability of *N. ampullaria* compared to the other species (Supplementary Table S3). The PLS-DA model was well-validated using a permutation test with p < 0.001 after 1000 permutations (Supplementary Fig. S1).

### Universal metabolite response to temperature stress

**Figure 3:**
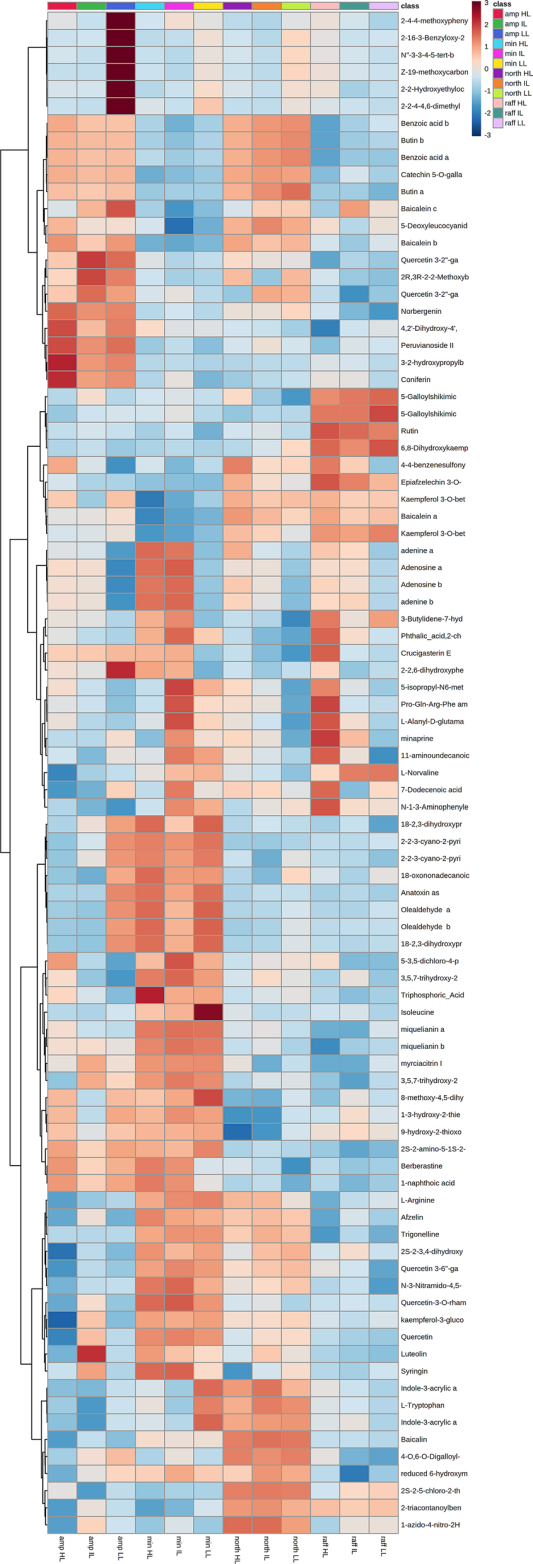
Heatmap of 89 identified metabolites from the 4 *Nepenthes* species based on Euclidean distances and Ward clustering. The metabolites concentrations are represented on a log scale. A more detailed bar chart of the important metabolites is provided in the Supplementary Fig. S2.

**Figure 4:**
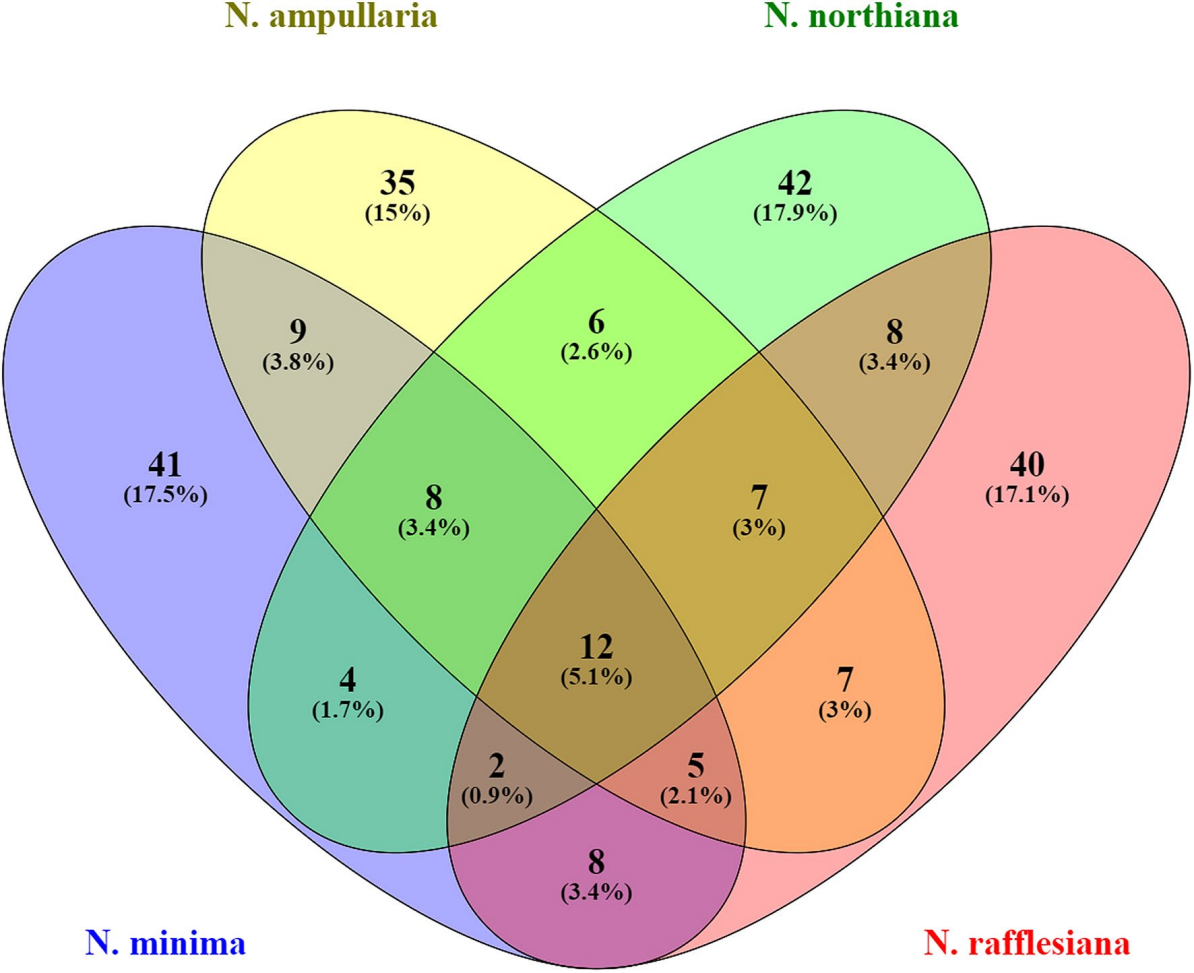
Venn diagram based on similarly expressed metabolites (highest expression recorded on either highland or lowland condition of the identified metabolites) among the 4 *Nepenthes* species in response to the three temperature conditions. Please refer to the supplementary file for the metabolites.

Our result showed that the metabolites were greatly affected by both lowland. Heat stress and highland cold stress conditions. While the metabolites expressed differed significantly among the four species (Fig. 3), high or lowland stress also led to a similar response in metabolite regulation across all our species (Fig. 4; Supplementary Table S4). Adenine, berberastine and 1-naphthoic acid were, for example, all expressed the highest under highland cold stress, whereas l-tryptophan (except *N*. *rafflesiana*), 18-oxononadecanoic acid, olealdehyde and indole-3-acrylic acid were all expressed the highest under lowland heat stress (Fig. 3; Supplementary Table S4). Interestingly, a flavone baicalein together with its isomers showed the highest accumulation at both highland and lowland conditions (Supplementary Table S4). Among the identified compounds, certain groups showed consistent expression among all the *Nepenthes* sp. such as purine bases (highest expression at highland condition), fatty acyls, amino acid, and indoles (highest expression level at lowland condition).

### Species-specific metabolite response to temperature stress

Our results also revealed unique combinations of metabolite responses towards heat and cold stress at interspecific level. For instance, *N*. *ampullaria* had the highest accumulation of alkaloid trigonelline and amino acid isoleucine under highland cold stress exposure while *N*. *minima* had them highest at lowland heat stress. *N*. *northiana* displayed high accumulation of norvaline under cold stress. Besides that, within the identified groups, most of the detected flavonoids had the highest expression under lowland conditions for *N. northiana* and *N*. *rafflesiana*, and highland condition for *N. minima*, while most of the flavonoids showed no change in response in *N. ampullaria*. Organic acid was recorded with the highest expression under highland conditions for *N*. *minima* and *ampullaria*, and lowland condition for *N*. *northiana*.

Apart from the universal and species-specific metabolite response mentioned above, we were able to observe metabolites accumulation patterns that were consistent for a subset of the species. For examples, under lowland heat stress, syringin exhibited the highest accumulation for *N. northiana* and *N. rafflesiana*, while adenosine exhibited the highest accumulation for *N. ampullaria*, *N. northiana* and *N. rafflesiana* under highland cold stress. A complete list of the metabolites expressed for each species under differing temperature conditions is provided in the Supplementary Table S4, and a more detailed bar chart of the important metabolites is provided in the Supplementary Fig. S2.

### Biosynthetic pathways and metabolic networks

**Figure 5:**
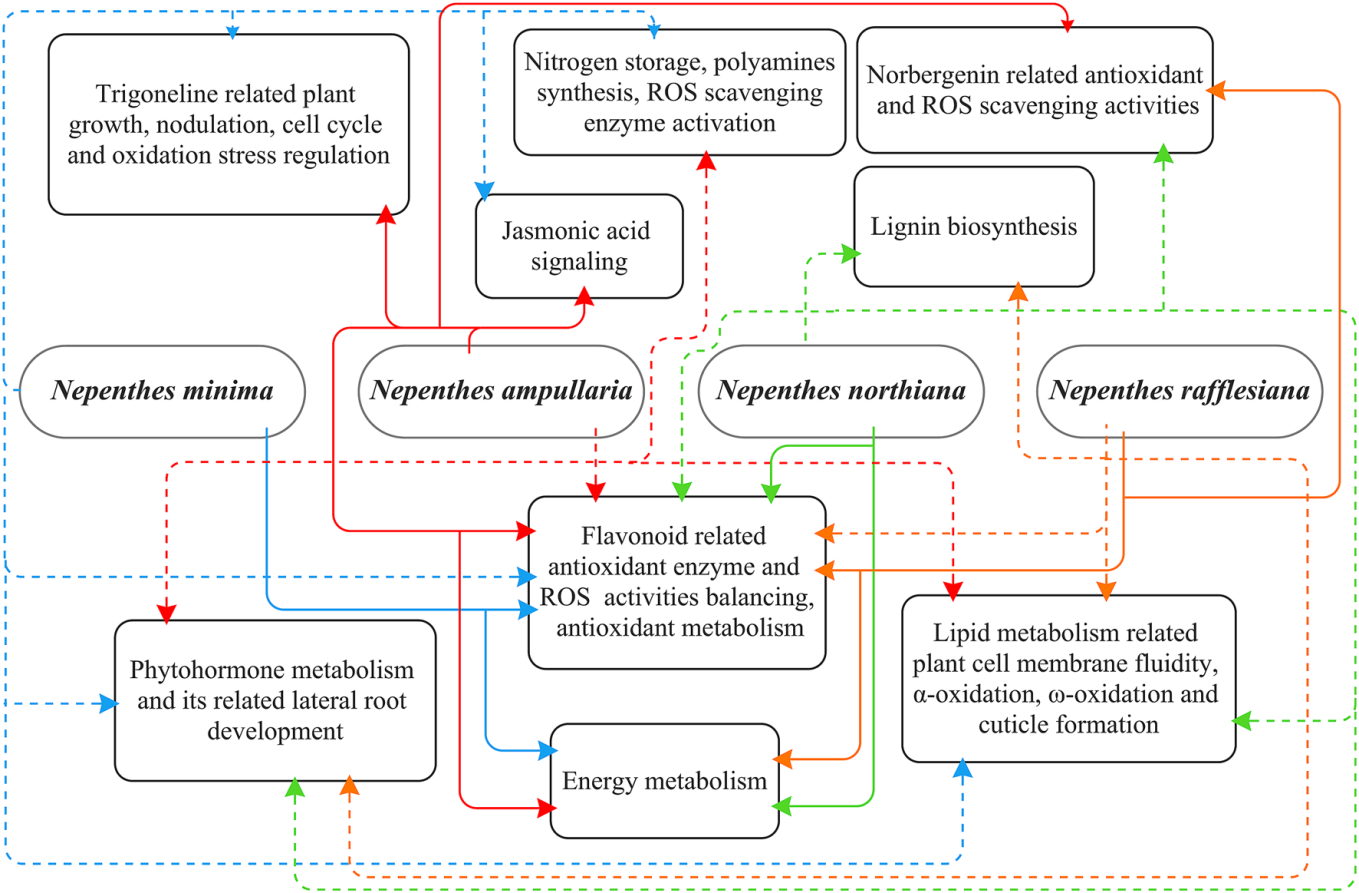
Adapting strategies applied by all 4 *Nepenthes* species in response to different environmental conditions. dot lines: strategies that used under highland cold stress exposure; solid lines: strategies that used under lowland heat stress exposure.

We identified 14 metabolites that are involved in 32 metabolic pathways, including biosynthesis of phenylpropanoid and flavones derivatives, flavonols, flavonoids, amino acids, secondary metabolites and lignins, as well as coniferin metabolism (Table 1). A metabolic network was created to summarize the major heat and cold stresses adapting strategies found in the 4 *Nepenthes* species (Fig. 5).

## Discussion

Till today, we have no clear understanding on the adaptation or survival mechanisms acquired by the different *Nepenthes* species to their climatic conditions. The distinctiveness of *N*. *ampullaria* and *N*. *rafflesiana* (capable to inhabit lowland and highland altitudes), and *N*. *minima* (unique highlander that is able to grow at lowland conditions), as well as *N*. *northiana* from an extreme habitat (limestone vegetation), made them the target species for the present study. We aimed to shed light on their unique adaptation mechanisms by studying their metabolomes in response to different environmental conditions. For the purpose of this study, we considered temperature to be the main environmental difference between highland (with temperatures down to 8 °C during the night) and lowland (up to 33 °C during the day) and exposed all four *Nepenthes* to temperatures representative of lowland, intermediate and highland conditions. *Nepenthes* are known to produce a wide range of metabolites but their metabolites have so far only been studied for their enzymatic properties and pharmacological potential^26–28^. The role of metabolites as regulatory/signaling agents, or in defense against biotic and abiotic stress (such as temperature changes), has been described in other plant species such as *Arabidopsis*, *Triticum*, and *Vitis*^29–31^. It is important to note that no additional experimental validation of the candidates has been carried out in this study. Nonetheless, our study provides the first insight into the response (at metabolome level) of *Nepenthes* plants originating from different climatic niches to temperature stress.

### Universal adaptation strategy

Plants possess various acclimatization strategies to survive temperature stresses, which includes the accumulation of flavonoids, alterations in the membrane lipid composition and signaling, phytohormones regulation and signaling, increased in transpiration, ROS scavenger accumulation and many more^32–40^. Our results indicate that some of these strategies are important in *Nepenthaceae* as well (Figs. 2, 3, 5).

Under lowland high temperature condition, *Nepenthes* seems to overcome the heat stress by increased phytohormone metabolism and related lateral root development. l-tryptophan is known to be involved in auxin indole-3-acetate synthesis^41,42^, while isoleucine is known to be the key amino acid that activates endogenous phytohormone jasmonic acid^43^. Phytohormone auxin was previously recorded to be positively affected by heat stress^44–46^ and is responsible for plant root formation^47,48^. We hypothesize that the observed increased auxin metabolism might increase lateral root development^48^. This would potentially contribute to a reduction in water loss caused by increased transpiration, and provide a cooling effect for the plant. Similar strategies have been found in *Arabidopsis thaliana*, where high temperature increased water loss via transpiration and enhanced leaf cooling capacity^49^. Besides that, the importance of α-oxidation (olealdehyde) and ω-oxidation (18-oxononadecanoic acid) seem to play a role in the response to heat stress in *Nepenthes* plants as well. Both oxidation processes, which involve aldehyde dehydrogenase as the key enzyme, are known to yield unsaturated fatty acid (α-oxidation) and dicarboxylic fatty acid (ω-oxidation) in which the unsaturated fatty acid is responsible in maintaining the fluidity of plant cell membrane lipids and dicarboxylic acid is essential for the cuticle formation in plant^50–53^. A similar increased expression of aldehyde dehydrogenase genes was also observed in *Arabidopsis* plants under heat stress exposure^54^. Interestingly, the amino acid norvaline was previously recorded in cold and drought stress responses^29,55^ and showed a universal contribution in our data (Fig. 3; Supplementary Table S4).

Our results highlighted the potential utilization of energy metabolism (ATP) by *Nepenthes* plants to overcome the stress caused by exposure to low temperature. Both adenine and adenosine, which were expressed the highest level under cold condition, are known for their importance for proper functioning of plant cell, nucleic acid synthesis and energy metabolism^56,57^. Similar effects were also observed in other plants. For example, increase of energy in the form of ATP was recorded during the cold acclimation of winter rape plants when temperatures dropped down to 5 °C^58^ and winter weeds (*Triticum aestivum* L. cv. Frederick) when temperatures went down to 2 °C^59^. The authors suggested the possibility of the energy utilization through the accumulation of ATP during the initial plant acclimation, to synthesize glycoprotein nature or other protective substances of protein that could increase the cold hardiness of the plant membranes^59^. Besides plants, similar ATP increasement under cold conditions has been reported in ice worms (*Mesenchytraeus solifugus*)^60^, psychrophilic microorganisms^61^ and bacteria^62^ with strong evidence of a linear relationship between intracellular ATP concentration and cold tolerance^63^.

Like any other plant, an increased ROS scavenging activity via the secondary ROS scavenging system flavonoids seems to be applied by *Nepenthes* plants in defense against oxidative damage induced by heat and cold stresses. Flavonoids have been recorded to play different roles in different plants under one genus^64^, and sometimes one flavonoid may have different responses in a single plant species with different origin^65^. Our study showed distinctive flavonoid manipulation of the 4 *Nepenthes* species in response to the different environmental conditions provided. Among the identified flavonoids, our results highlight the distinctly different expression levels of baicalein isomers which could indicate different strategies of the species to overcome the environmental stress. Our results are supported by several other studies^66–69^, in which different flavonoid isomers resulted in various antioxidant activities. Together with its glucuronide form baicalin, both flavonoids play important roles in the balance between antioxidant enzyme and ROS activities in adaptive responses to temperature stress^70^. Limited studies are available on the role of proanthocyanidin in response to cold stress, however, An et al.^71^ have shown the importance of the R2R3-MYB transcription factor MdMYB23 in proanthocyanidin accumulation under cold temperature (4 °C) exposure in apple (*Malus* × domestic). Our data lends further support to the involvement of proanthocyanidin (Epiafzelechin 3-*O*-gallate-(4beta- > 6)-epigallocatechin 3-*O*-gallate) in response to cold stress.

### Species-specific adaptation strategies

The metabolite regulation patterns showed that responses to temperature changes are linked to the plant habitats. Thermal specialization in tropical plant species indicate further inabilities for highland *Nepenthes* plants to adapt to changing environments^72–74^. The pyrophytic species *Nepenthes minima*, however, is unique as it adapted well in the greenhouse under lowland conditions. Its habitat is known to experience high temperatures up to 38 °C and prone to seasonal burning, with re-growing observed from the plant rootstock after the wildfires^75^. This could be the reason the species developed heat tolerance. Our temperature metabolome study revealed the ability of this species to manipulate production of amino acids and phytohormones in their heat stress adaptation. We discovered that this highland species possesses the same heat adaptation strategy like the lowlander *N*. *rafflesiana*, such as increased nitrogen storage, and polyamides synthesis via l-arginine accumulation^76^. Nitrogen storage is known to be crucial in heat shock protein production which plays a vital role in surviving heat stress^77^, while polyamines and l-arginine play a major role in activating ROS-scavenging enzymes under abiotic stress^76,78^.

Apart from the increased production of the phytohormone auxin observed in all 4 *Nepenthes* species studied, *N*. *minima* upregulated the production of two other phytohormones: jasmonic acid and trigonelline. The importance of the endogenous phytohormone jasmonic acid for heat and cold toleration in plants have been previously recorded^44,79,80^. Trigonelline was previously linked with various regulatory roles in relation to plant cell cycle regulation, nodulation, oxidative stress, as well as the growth of the plant^81–83^. Interestingly, a similar manipulation of the two hormones can also been observed in the lowlander *N*. *ampullaria*. However, instead of the lowland condition, *N*. *ampullaria* up-regulated their production under highland cold stress.

As a lowlander, *N*. *ampullaria* was found to be capable of inhabiting a wide altitude range- including highland environments (up to 2100 masl^3^). That is to say, the species even as a lowlander, is capable of tolerating low temperatures. Besides the two phytohormones mentioned above, we also observed an increased production of norbergenin, which possesses both antioxidant and ROS scavenging activities. It is likely that *N*. *ampullaria* uses it to protect themselves from cold stress induced oxidative damage^33,34^. A similar potential protection strategy was also observed in *N*. *rafflesiana*, which has been recorded growing at 1500 masl according to Adam et al.^84^.

While two metabolites involved in lignin biosynthesis pathways (coniferin and syringin) were detected in all four species, two of the lowland species (*N. northiana* and *rafflesiana*) displayed significantly higher accumulation of syringin under lowland conditions. *N. northiana* is commonly found on limestone hills, a harsh environment composed of calcium carbonate, alkaline pH and highly susceptible to drought^85^, while *N. rafflesiana* can be found in open habitats such as degraded, dry laterite and podsols^4,86^. In Matang, Kuching, Sarawak, Malaysia, *N. rafflesiana* has also been observed in open areas with direct exposure to sunlight and heat (anecdotal observations). Based on our data, it seems that in response/adaptation to the sun, heat, and drought, both *N. northiana* and *rafflesiana* developed a self-protection strategy by increasing lignification to inhibit water loss from plant tissue^87^. Similar adaptation mechanisms have been shown for Norway spruce, *Ctenanthe setosa*, and wheat^88–90^.

### Survival in a changing climate

Past studies revealed the importance of ecological adaptation of *Nepenthes* as the key determining factor driving, not only the diversification of pitcher morphology and their prey trapping mechanisms, but also the evolution of plant nutrient sequestration strategies^91,92^. In this study, we observed significant changes in the individual metabolomes of four *Nepenthes* species towards high and low temperature heat stress. Some pf the observed responses, such as the lignification, are verily likely linked to their habitat niches (Fig. 5).

*Nepenthes* are known to be susceptible towards climate change. Due to the narrow endemism geographical distribution of certain species, especially some highlanders that are confined to single mountain summits, they are at particularly high risk of species extinction^92,93^. Previously ecological niche modeling and maxent modeling have determined the climatically suitable area (habitat) for some species such as *N*. *rafflesiana*, *N*. *tentaculate*, *N*. *macrophylla* and *N*. *lowii*, via application of the climatic (such as rainfall and temperature) and edaphic (such as landform, soil association, soil parent material and soil suitability) variables^93,94^. The present eco-metabolomic study has highlighted the flexible responses (in terms of metabolite production) of the plant genus to adapt to environmental heat and cold stress. Our data does suggest that some lowlander species are indeed able to produce metabolites required to deal with increased high temperature stress. Hence, the future for selected species might not be as bleak as predicted.

## Conclusion and final remark

Our eco-metabolomic study on the impact of lowland heat stress and highland cold stress revealed different metabolic fingerprints and potential adaptation strategies based on the species ecological niches. Our study demonstrated both universal (shared across all four species studied) and species-specific responses increase in selected metabolites under heat and cold stress. The metabolites found indicate the importance of several adaptation strategies ranging from increased ATP and ROS production, to the potential increased root development via auxin production. Lastly, we suggest more studies on plant metabolomes to achieve a better understanding of the adaption of *Nepenthes* (and other plant) species to their habitats.

## Methods

### Nepenthes

Four *Nepenthes* species namely *Nepenthes minima*, *Nepenthes ampullaria*, *Nepenthes northiana* and *Nepenthes rafflesiana* were pre-adapted at lowland greenhouse for a period of 6 months, at least, before subjected into climatic chamber with control environment conditions. The *Nepenthes* in this study represented highland and lowland climate conditions (Table 2; for morphological details of the plant, please refer to Jebb and Martin^3^ and Adam et al.^84^). We hypothesized that distinct metabolite regulation patterns can be distinguished between the *Nepenthes* species due to their adaptation towards different geographical and altitudinal distribution.

**Table 2:** Four *Nepenthes* species employed in this study.

*Nepenthes* (*N.*) species	Habitat	Altitudinal distribution	Environmental niche assigned
*N. northiana*	Limestone hills	0–500	Lowlander
*N. rafflesiana*	Open area, shady forest, offshore	0–1500	Lowlander
*N. ampullaria*	Damp, shady forest, swamp forest	0–2000	Lowlander
*N. minima*	Open grassland, with grey-yellow clay as substrate (highland grasslands of Central Sulawesi (Celebes)	1000–1700 with most localities lying above 1400 masl	Highlander

### Plant materials and growth conditions

All plants were grown in a Pol-Eko-Aparatura climatic chamber with phytotron system (Model: KK 750 FIT P) in a mixture of cocopeat and perlite (at a ratio of 10:0.5; g/g). All the plants were grown under a 12 h light and 12 h dark photoperiod.

Plants were exposed to (a) lowland (33 °C day/28 °C night), (b) intermediate (25 °C day/18 °C night), and (c) highland conditions (23 °C day/8 °C night) for 7 days before being harvested. The growing tips, the active growing part of the plants, were harvested and freeze-dried (Labconco Freezone 6 Freeze Dryer System), and metabolites extracted.

### Sample preparation

One (± 0.1) mg freeze-dried plant samples were weighted, ground, and exhaustedly extracted with 600 µL of solvent mixture of methanol:chloroform:ultrapure water (with resistance of 18.2 Ω cm^−1^) with 1% sodium chloride added (1:1:1 v/v/v). Mixtures were vortexed for 30 min at room temperature, followed by 30 min centrifugation at 3000 × *g* maintained at 4 °C. The lower layer was then transferred into a new borosilicate tube and vacuum dried using a speed concentrator. Dried extracts were then reconstituted using 400 µL of methanol and filtered using 0.2 μm PTFE membrane filter before subjected to liquid chromatography and mass spectrometry analysis.

### Metabolome profiling

The extracted samples were profiled based on previously published method^95^. Briefly, 10 µL of the samples were injected into Kinetex F5 (2.1 × 100 mm, 2.6 μm; Phenomenex, Torrance, CA, USA) for chromatographic separation via Vanquish™ Horizon UHPLC system (Thermo Fisher Scientific, USA). During analysis, the column was maintained at 40 °C with the flow rate of 600 µL/min. The mobile phase was composed of 2 solvents; solvent A (H_2_O—0.1% HCOOH—1% 10 mM NH_4_OAc) and solvent B (acetonitrile/methanol [6:4 v/v]—1% of 0.1% HCOOH—1% 10 mM NH_4_OAc). The gradient elution program was initiated from 1 to 40% solvent B in 5 min, followed by 100% solvent B from 5.1 to 8 min and maintained for 2 min. Before injecting the next sample, the initial gradient was employed to condition the column for 3 min. UHPLC system was coupled with electrospray ionization Impact II QToF-mass spectrometry system (Bruker Daltonic, Germany). Mass-to-charge ratio (*m/z*) was set between 50 and 1500 for data acquisition. The heated electrospray ionization (ESI) was deployed at 4200 V for positive. Ion source gas temperature and flow rate was set at 300 °C and 12 L/min, respectively.

Mass calibration solution, 10 mM sodium formate was introduced post-column through a 6-port valve diverted between 0.1 and 0.3 min. Acquired *m/z* was calibrated against the introduced sodium formate, and then subsequently converted into a mzXML file format.

### Metabolomics data processing

Raw data was exported in .mzXML format prior to MZmine 2 analysis^96^. The software provides noise filtering, peak detection, alignment, normalization, alignment, and gap-filling and exported data in .csv format. Exported .csv files were used for multivariate analyses with MetaboAnalyst 4.0^97^. Metabolite features with missing values > 45% were removed, and missing values imputed using K-nearest neighbors^98^. The data was log transformed and pareto scaled. Metabolite features (ANOVA *p* < 0.01) between the 4 *Nepenthes* species under 3 environmental conditions further underwent compound matching and analysis. The .csv file with significantly changed metabolite features is provided in the supplementary file. The current analysis focused on polar the layer only as the non-polar layer demonstrated no significant difference (data excluded). All statistical analyses were performed on the positive ion data sets.

### Metabolite annotation and identification

Metabolite features, including accurate *m/z*, possible chemical formula, and the fragmentation pattern, were queried against biological databases (highest priority was given to the database KEGG, followed by PubChem, and the others such as ChEBI and ChemSpider) using in silico fragmenter MetFrag^99^. The candidate was chosen based on the following criteria: (a) highest score with at least 80% match of the major fragment ions towards the databases (b) lowest relative mass deviation error when compared to the theoretical value (c) lowest relative mass deviation error from the fragment ions matched. To increase the accuracy of the identified metabolites, we cross checked the matched compound with earlier literature on similar compound especially in *Nepenthes* or in other plants. Pathway Tools Omics Viewer, developed by the Plant Metabolic Network (PMN), was used to identify highly correlated metabolites and to visualize the biosynthetic pathways^100^.

### Statistical analyses

Multiple comparison of mean tests, bar chart and pie chart were performed using Microsoft Excel. The data were pre-transformed using generalized logarithm transformation method via MetaboAnalyst 4.0. A two-way ANOVA with Tukey’s Post hoc analysis performed using PAST software^101^. Multivariate analyses including analysis of variance (ANOVA), partial least squares–discriminant analysis (PLS-DA), hierarchical cluster analysis and heat map were performed using MetaboAnalyst 4.0^97^. Venn diagram was created using Venny 2.1-developed at Bioinformatics for Genomics and Proteomics (BioinfoGP)^102^. Correlation values of the highly correlated metabolites with the biosynthetic pathways was performed using Omics Viewer^100^. Figure 5 was constructed using ConceptDraw OFFICE 6^103^.

## Supplementary Information

Supplementary Information 1.

Supplementary Information 2.
