# Supplementary material for: A comparative UHPLC-Q/TOF–MS-based eco-metabolomics approach reveals temperature adaptation of four Nepenthes species
Source: Sci Rep. 2020 Dec 14;10:21861. doi: 10.1038/s41598-020-78873-3 (PMC7736350; doi:10.1038/s41598-020-78873-3)
Supplement: Supplementary file 2 — Supplementary Information 2. [file 41598_2020_78873_MOESM2_ESM.doc]

# Supplementary material

# A Comparative UHPLC-Q/TOF-MS-Based Eco-Metabolomics Approach reveals Temperature Adaptation of Four *Nepenthes* Species

**Changi Wong^1^, Yee Soon Ling^2^, Julia Lih Suan Wee^3^, Aazani Mujahid^4^ & Moritz Müller^1*^**

^1^Faculty of Engineering, Computing and Science, Swinburne University of Technology Sarawak, 93350 Kuching, Malaysia.

^2^Water Research Unit, Faculty of Science and Natural Resources, University Malaysia Sabah, 88400 Kota Kinabalu, Sabah, Malaysia**.**

^3^Faculty of Resource Science and Technology, Universiti Malaysia Sarawak, 93400 Kota Samarahan, Sarawak, Malaysia

^4^Institute of Biodiversity and Environmental Conservation, Universiti Malaysia Sarawak, 94300 Kota Samarahan, Sarawak, Malaysia

Corresponding author: Moritz Müller, [mmueller@swinburne.edu.my](mailto:mmueller@swinburne.edu.my)


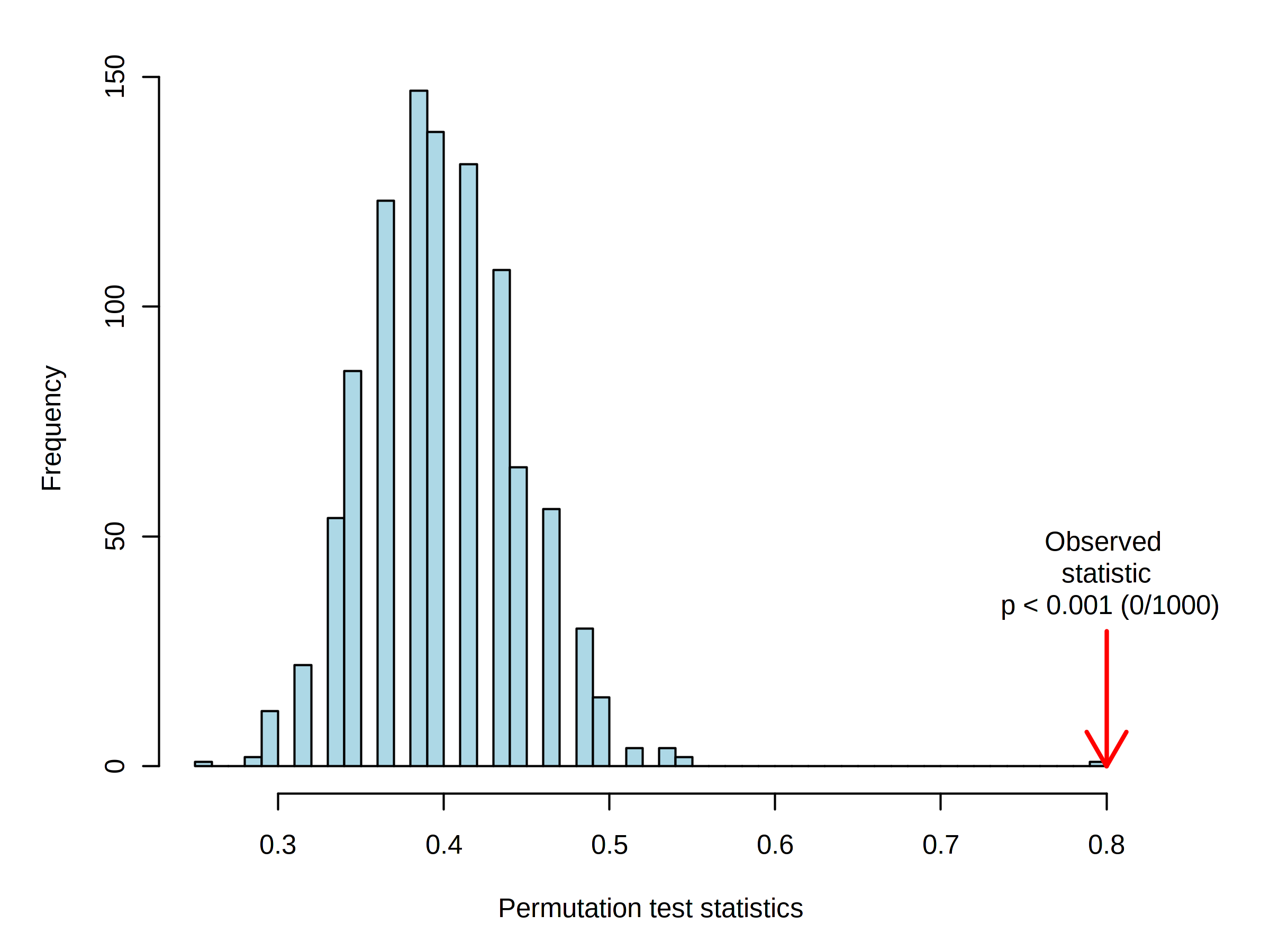


**Figure S1.** Permutation test for model PLS-DA model validation.


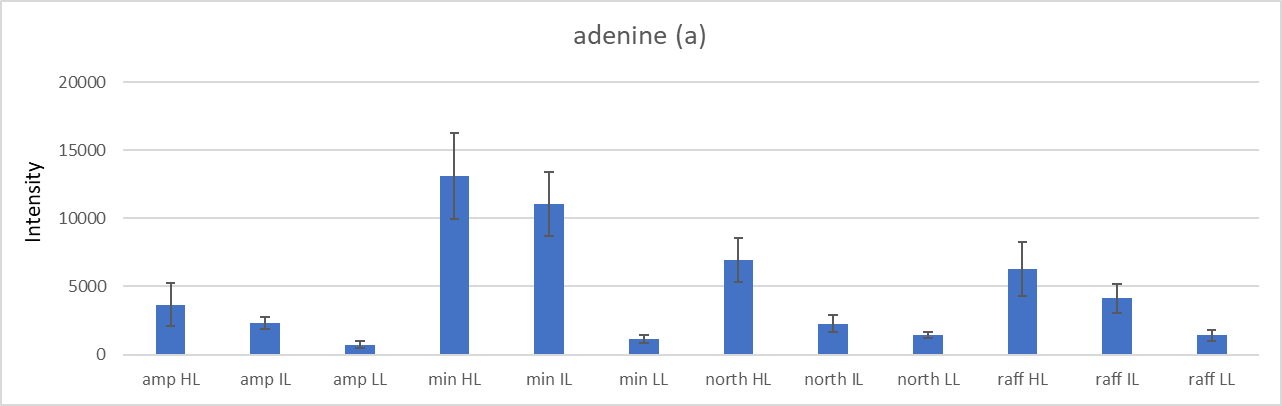

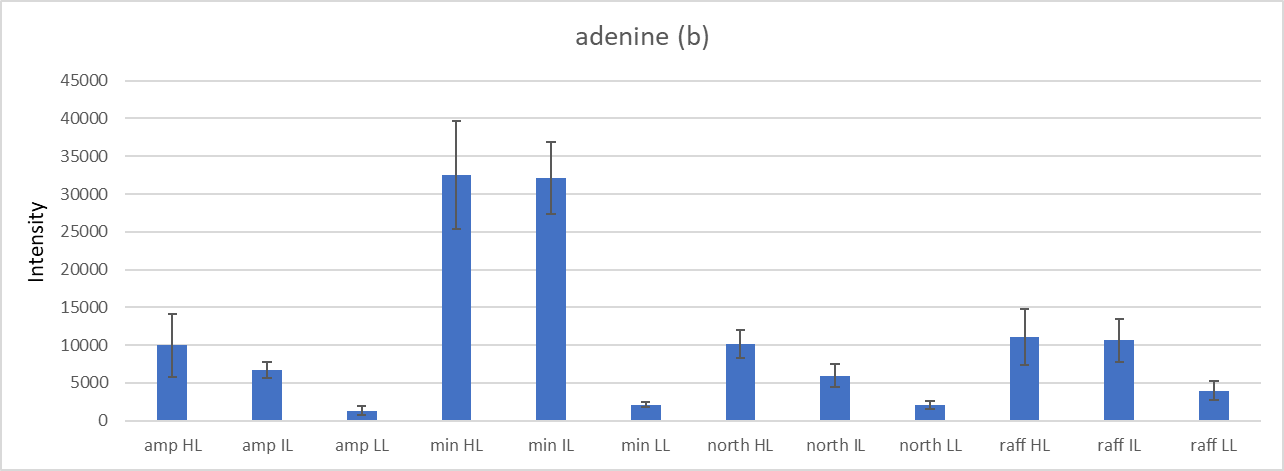

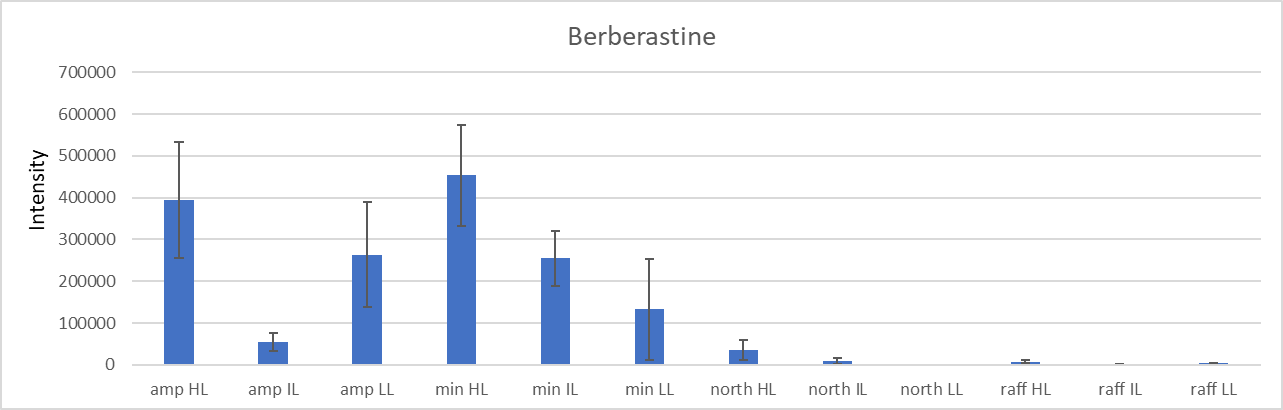

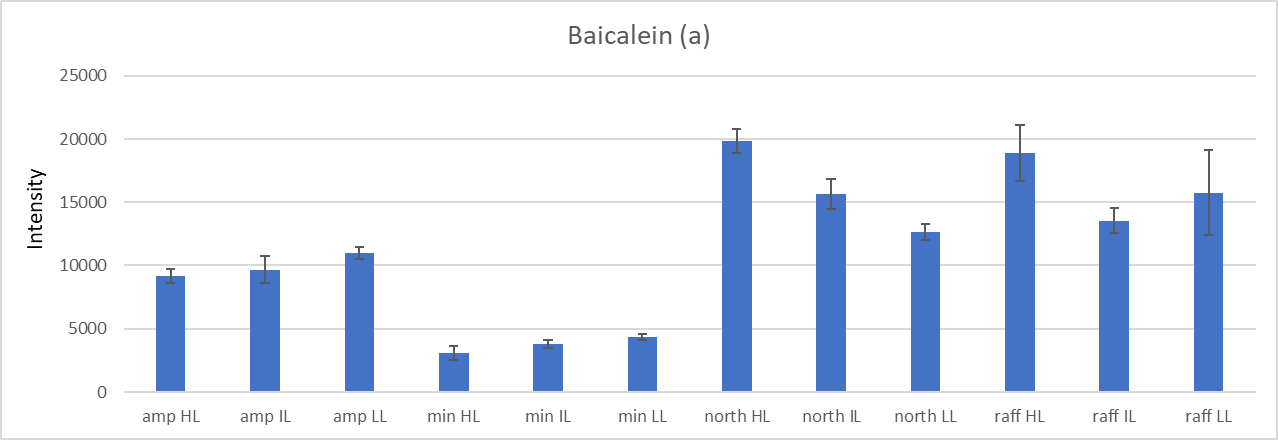

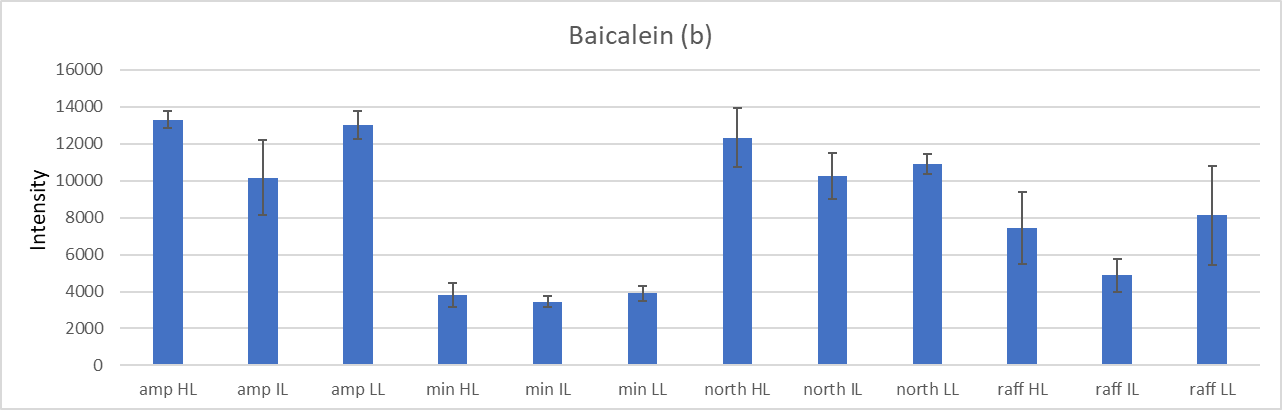

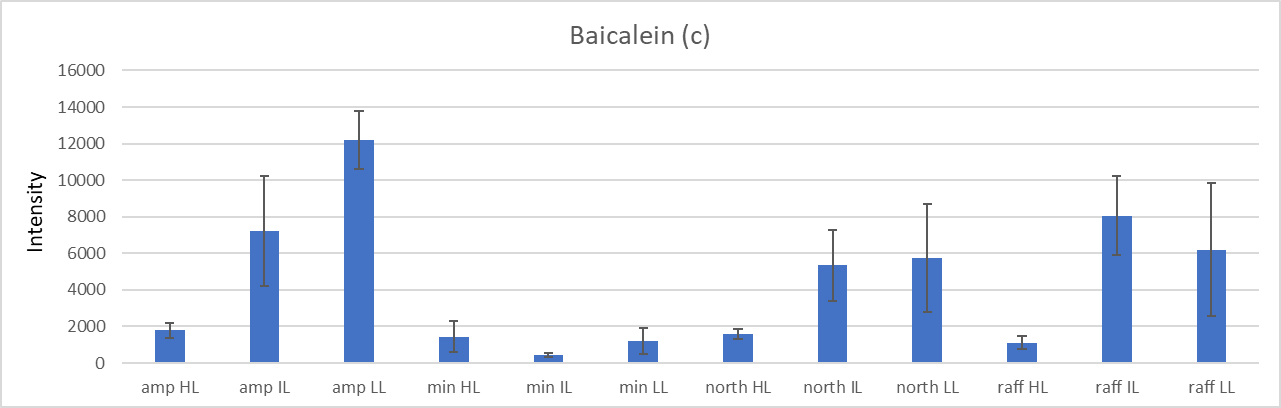

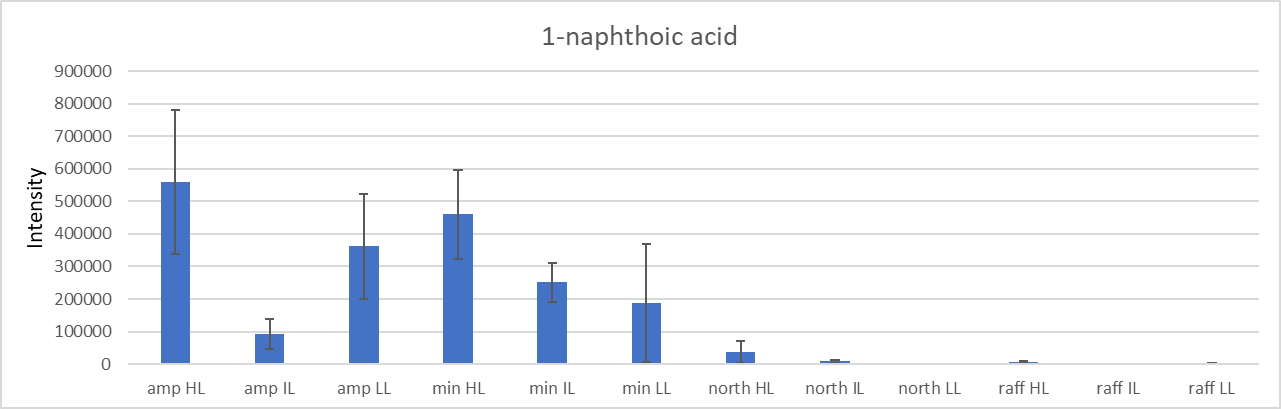

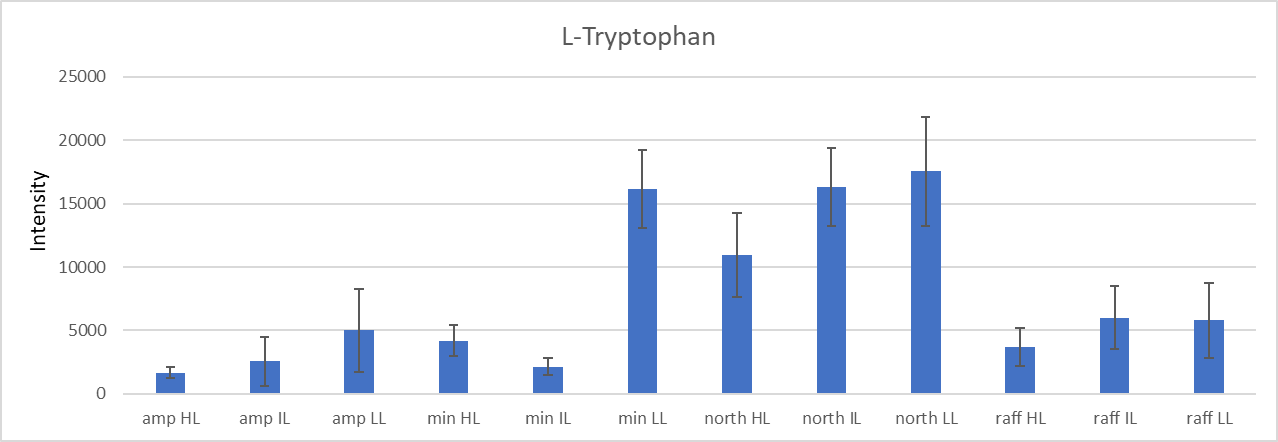

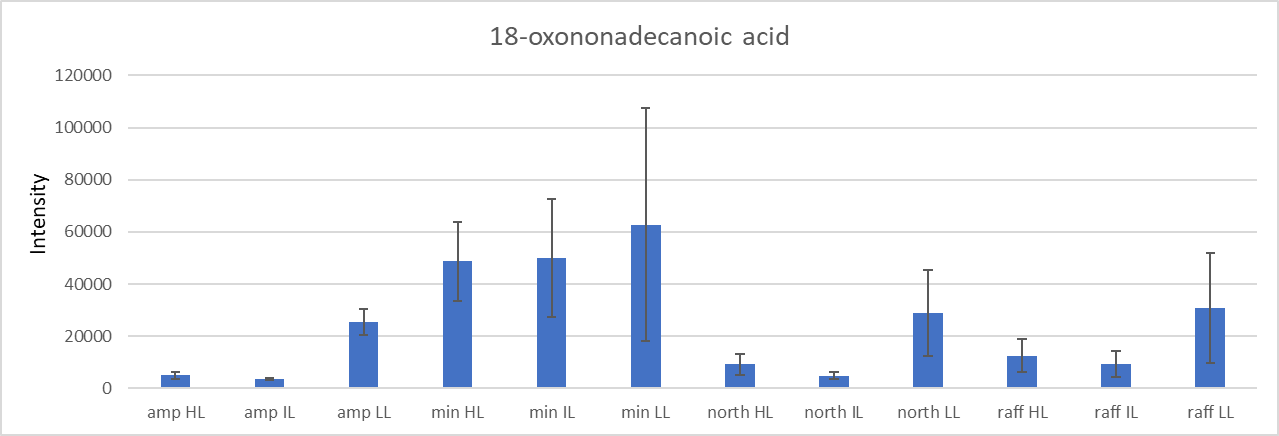

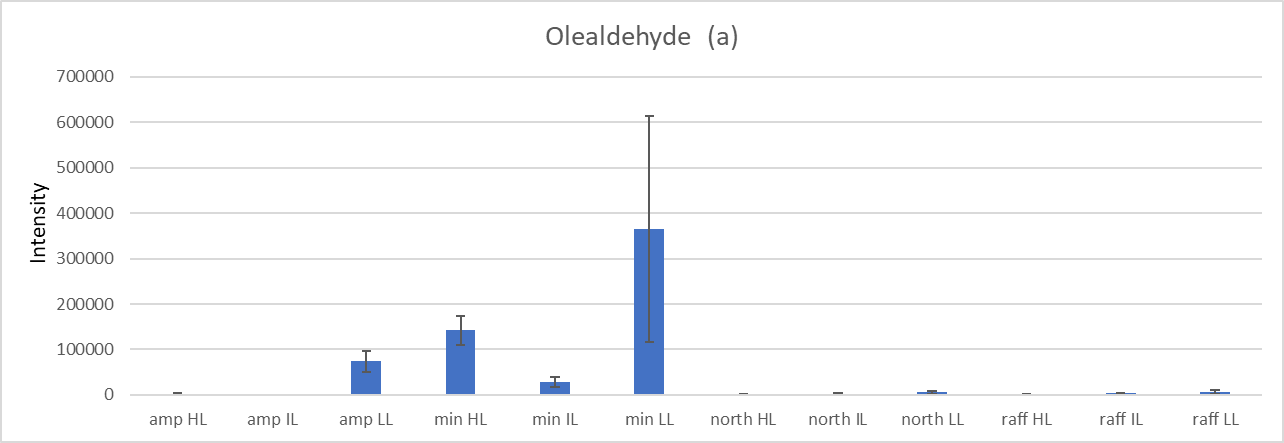

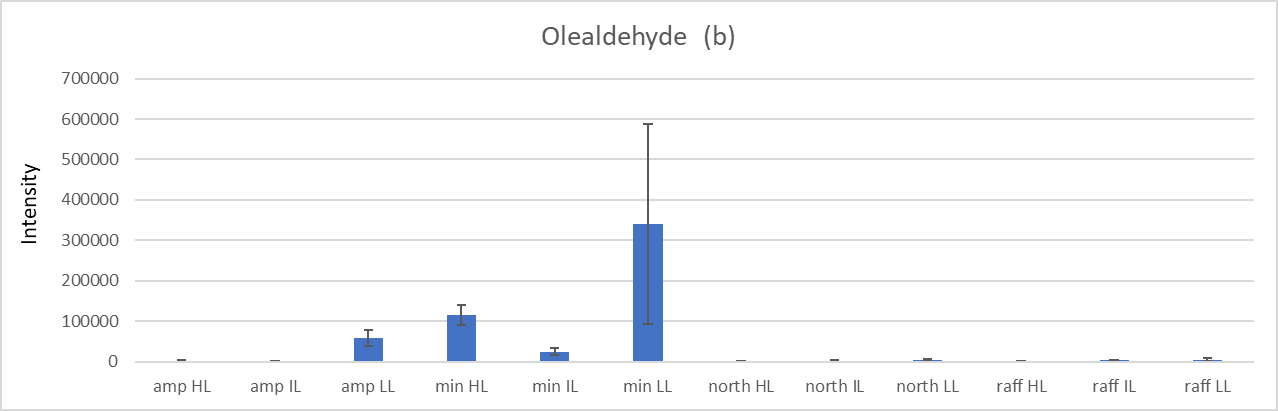

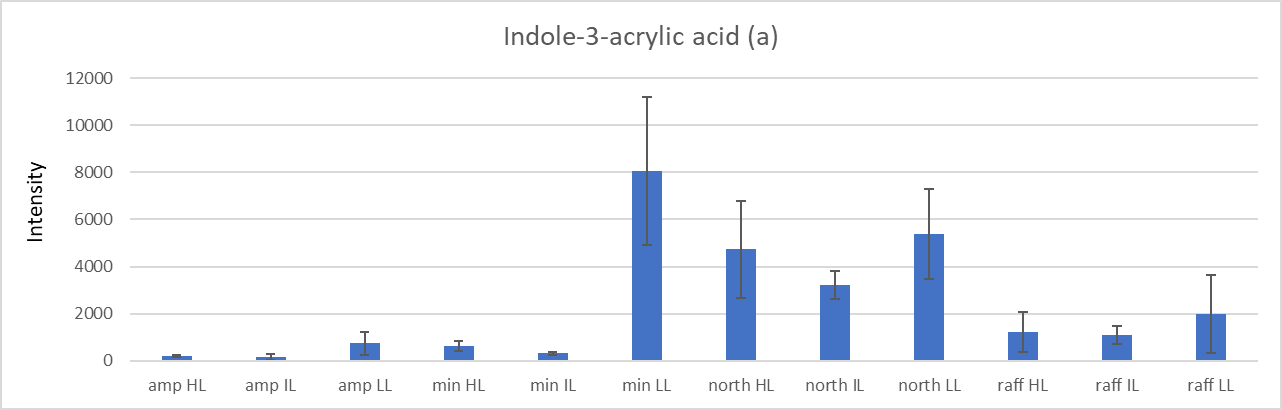

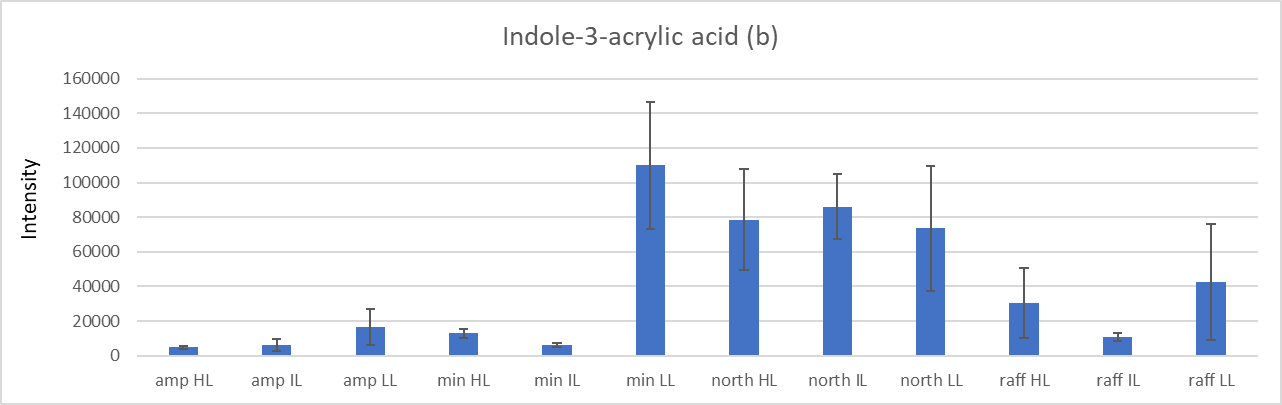

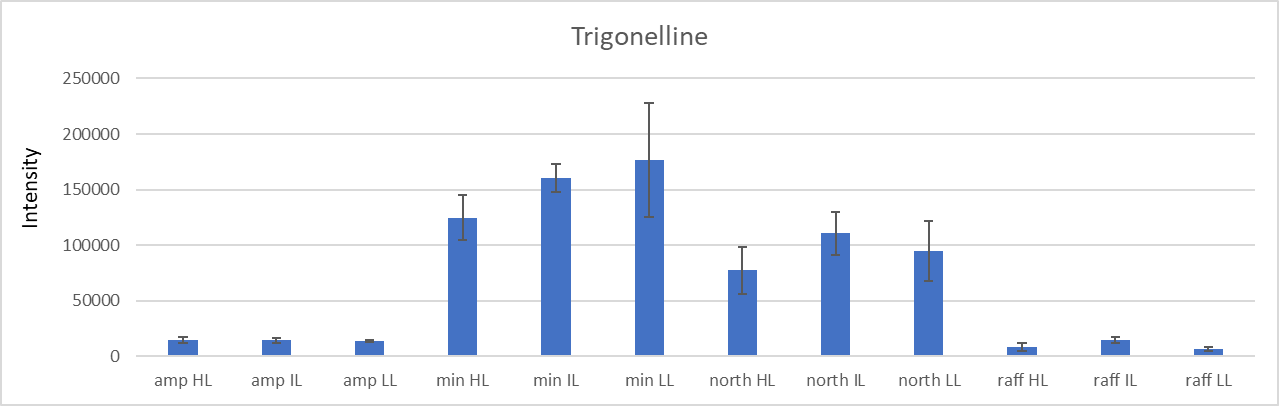

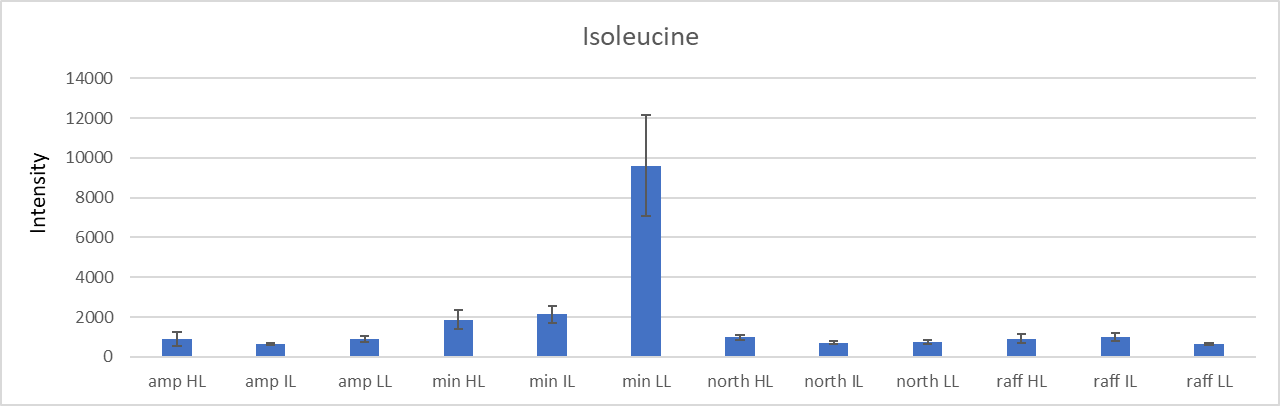

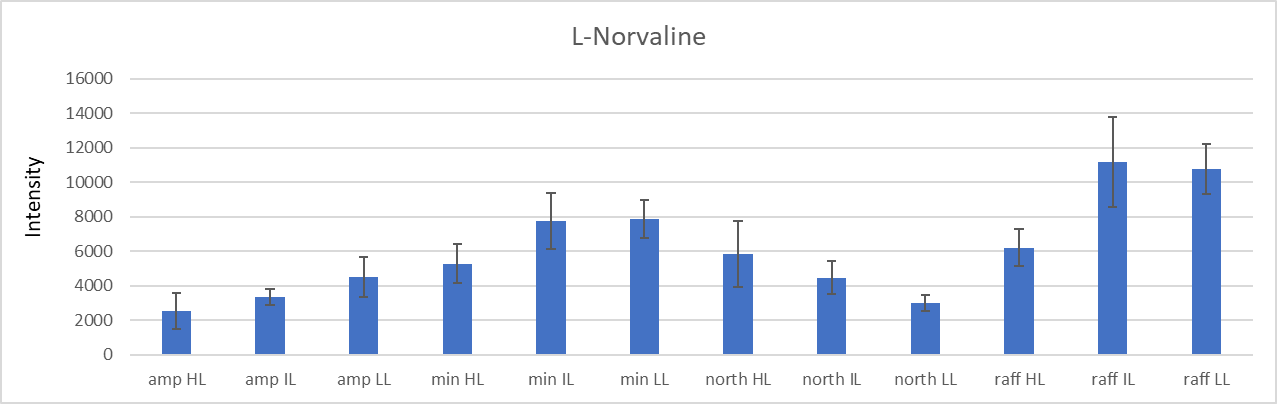

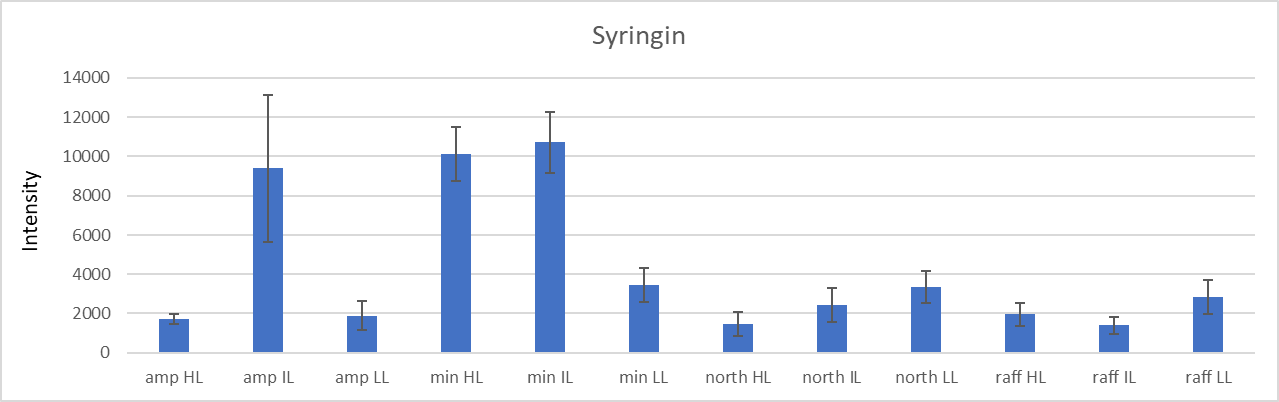

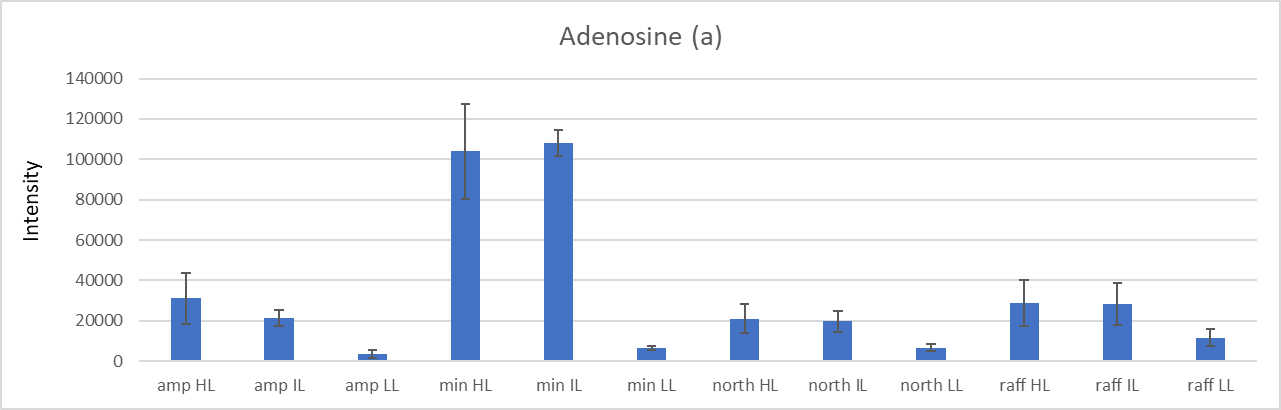

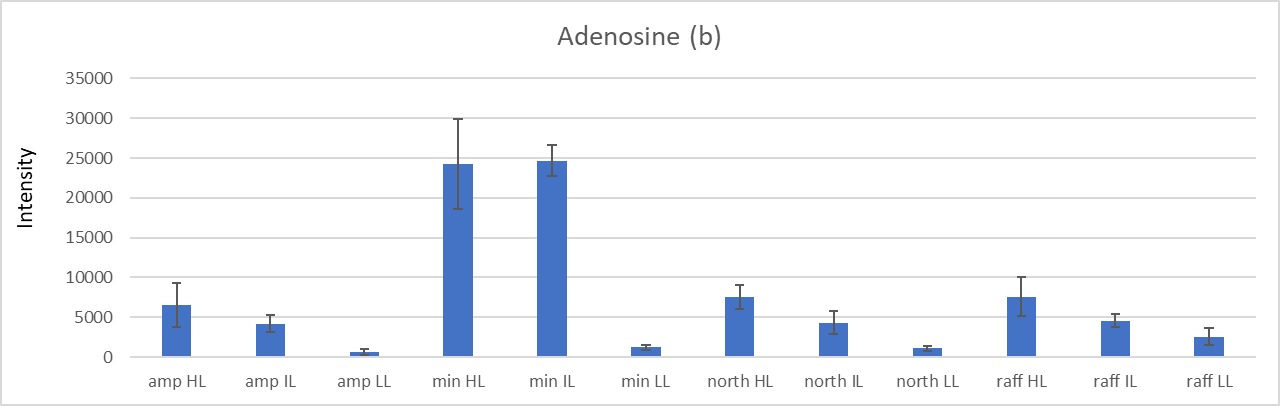


**Figure S2.** Bar charts of select metabolites that were elevated in different growth conditions and *Nepenthes* (*N*.) species. n = 5. Error bars represent the standard error. Abbreviations: amp represents *N*. *ampullaria*, min represents *N*. *minima*, north represent *N*. *northiana*, and raff represents *N*. *rafflesiana*. HL represent highland condition, IL represent intermediate condition, and LL represent lowland condition.

**Table S1. Identification of the 125 significantly altered metabolic features.**

| **Compound Groups** | **No.** | **RT (min)** | **Adduct** | **Mass** | **Tentative Identification** | **Formula** | **Monoisotopic Mass** | **PPM (error)** | **Database ID** |
| --- | --- | --- | --- | --- | --- | --- | --- | --- | --- |
| Flavanoids | 19 | 2.17 | [M + NA]+ | 471.0905 | Quercetin 3-O-rhamnoside | C_21_H_20_O_11_ | 448.101 | 0.62 | C01750 |
|  | 135 | 2.14 | [M + H]+ | 449.1091 | Kaempferol-3-glucoside | C_21_H_20_O_11_ | 448.101 | 1.83 | C12249 |
|  | 149 | 1.94 | [M + H]+ | 291.085 | 5-Deoxyleucocyanidin | C_15_H_14_O_6_ | 290.079 | -4.31 | C09736 |
|  | 165 | 1.98 | [M + H]+ | 617.1169 | Quercetin 3-(6''-galloylglucoside) | C_28_H_24_O_16_ | 616.106 | 5.81 | CID 44259190 |
|  | 172 | 1.91 | [M + NH4]+ | 316.2121 | Minaprine | C_17_H_22_N_4_O | 298.1794 | -3.79 | CHEBI:51038 |
|  | 277 | 2.02 | [M + H]+ | 443.0981 | Catechin 5-O-gallate | C_22_H_18_O_10_ | 442.09 | 1.85 | CID 15689618 |
|  | 312 | 2.12 | [M + H]+ | 303.0503 | Quercetin | C_15_H_10_O_7_ | 302.0427 | 1.06 | C00389 |
|  | 394 | 2.2 | [M + NA]+ | 485.1431 | 4,2'-Dihydroxy-4',6'-dimethoxychalcone 4-glucoside | C_23_H_26_O_10_ | 462.153 | 1.88 | CID 13870531 |
|  | 406 | 2.21 | [M + NA]+ | 609.0862 | Quercetin 3-(2''-galloyl-alpha-L-arabinopyranoside) | C_27_H_22_O_15_ | 586.096 | 1.74 | CID 44259256 |
|  | 425 | 2.16 | [M + H]+ | 587.1048 | Quercetin 3-(2''-galloyl-alpha-L-arabinopyranoside) | C_27_H_22_O_15_ | 586.096 | 2.51 | CID 44259256 |
|  | 409 | 1.92 | [M + NH4]+ | 512.1772 | Peruvianoside II | C_23_H_26_O_12_ | 494.142 | 2.75 | CID 42608014 |
|  | 464 | 1.76 | [M + H]+ | 447.0904 | Baicalin | C_21_H_18_O_11_ | 446.0849 | -3.95 | C10025 |
|  | 541 | 12.77 | [M + H]+ | 271.0604 | Baicalein (a) | C_15_H_10_O_5_ | 270.0528 | 1.11 | C10023 |
|  | 548 | 2.02 | [M + H]+ | 271.0604 | Baicalein (b) | C_15_H_10_O_5_ | 270.0528 | 1.11 | C10023 |
|  | 591 | 1.82 | [M + H]+ | 271.0604 | Baicalein (c) | C_15_H_10_O_5_ | 270.0528 | 1.11 | C10023 |
|  | 542 | 12.77 | [M + NA]+ | 441.0802 | Kaempferol 3-O-beta-D-xyloside (a) | C_20_H_18_O_10_ | 418.09 | 2.39 | C20727 |
|  | 543 | 2.09 | [M + NA]+ | 441.0802 | Kaempferol 3-O-beta-D-xyloside (b) | C_20_H_18_O_10_ | 418.09 | 2.39 | C20727 |
|  | 550 | 2.01 | [M + NA]+ | 633.1449 | Rutin | C_27_H_30_O_16_ | 610.1534 | 3.67 | C05625 |
|  | 553 | 1.89 | [M + NA]+ | 649.1396 | 6,8-Dihydroxykaempferol 3-rutinoside | C_27_H_30_O_17_ | 626.148 | 3.75 | CID 44260049 |
|  | 588 | 2.34 | [M + H]+ | 883.1735 | Epiafzelechin 3-O-gallate-(4beta->6)-epigallocatechin 3-O-gallate | C_44_H_34_O_20_ | 882.164 | 2.55 | CID 14521010 |
|  | 128 | 2.24 | [M + NA]+ | 455.0961 | Afzelin | C_21_H_20_O_10_ | 432.106 | 2.11 | C16911 |
|  | 473 | 1.98 | [M + H]+ | 479.0828 | Miquelianin (a) | C_21_H_18_O_13_ | 478.075 | 1.09 | CHEBI:66395 |
|  | 500 | 2 | [M + NA]+ | 501.0653 | Miquelianin (b) | C_21_H_18_O_13_ | 478.0747 | 2.89 | CHEBI:66395 |
|  | 192 | 2.19 | [M + H]+ | 287.0556 | Luteolin | C_15_H_10_O_6_ | 286.0477 | 2.17 | C01514 |
|  | 278 | 2.05 | [M + H]+ | 273.0763 | Butin (a) | C_15_H_12_O_5_ | 272.068 | 3.68 | C09614 |
|  | 281 | 1.82 | [M + H]+ | 273.0763 | Butin (b) | C_15_H_12_O_5_ | 272.068 | 3.68 | C09614 |
|  | 427 | 2.04 | [M + NA]+ | 501.1375 | Myrciacitrin I | C_23_H_26_O_11_ | 478.1475 | 1.51 | CHEBI:66417 |
|  | 193 | 2.27 | [M + H]+ | 623.1018 | 3,5,7-trihydroxy-2-[3,4,5-trihydroxy-2-[2,3,4-trihydroxy-6-(3,5,7-trihydroxychroman-2-yl)phenyl]phenyl]chromen-4-one (a) | C_30_H_22_O_15_ | 622.096 | -2.38 | CID 57691813 |
|  | 280 | 2.13 | [M + H]+ | 623.1018 | 3,5,7-trihydroxy-2-[3,4,5-trihydroxy-2-[2,3,4-trihydroxy-6-(3,5,7-trihydroxychroman-2-yl)phenyl]phenyl]chromen-4-one (b) | C_30_H_22_O_15_ | 622.096 | -2.38 | CID 57691813 |
|  | 488 | 1.97 | [M + NA]+ | 745.1761 | (2S)-2-(3,4-dihydroxyphenyl)-8-[(2S,4R)-2-(3,4-dihydroxyphenyl)-7-hydroxy-5-[(2S,3R,4S,5S,6R)-3,4,5-trihydroxy-6-(hydroxymethyl)oxan-2-yl]oxy-3,4-dihydro-2H-chromen-4-yl]-5,7-dihydroxy-2,3-dihydrochromen-4-one | C_36_H_34_O_16_ | 722.185 | 2.62 | CID 6325552 |
| Alkaloids | 15 | 0.47 | [M + H]+ | 138.0549 | Trigonelline | C_7_H_7_NO_2_ | 137.0477 | 2.80 | C01004 |
|  | 209 | 2.44 | [M + H]+ | 253.1074 | Anatoxin a(s) | C_7_H_17_N_4_O_4_P | 252.0987 | 5.63 | C19998 |
|  | 577 | 2 | [M + NA]+ | 375.1056 | Berberastine | C_20_H_18_NO_5_ | 352.118 | -4.35 | CHEBI:3065 |
| Amino acids | 158 | 0.5 | [M + H]+ | 175.118 | L-Arginine | C_6_H_14_N_4_O_2_ | 174.1117 | -5.74 | C02385 |
|  | 162 | 1.53 | [M + H]+ | 205.0977 | L-Tryptophan | C_11_H_12_N_2_O_2_ | 204.09 | 2.06 | C00078 |
|  | 228 | 0.47 | [M + H]+ | 132.1018 | Isoleucine | C_6_H_13_NO_2_ | 131.095 | -3.66 | C00407 |
|  | 242 | 0.47 | [M + H]+ | 118.0835 | L-Norvaline | C_5_H_11_NO_2_ | 117.079 | -23.74 | C01826 |
| Fatty Acyls | 155 | 3.63 | [M + H]+ | 199.1694 | 7-Dodecenoic acid | C_12_H_22_O_2_ | 198.162 | 0.61 | CID 5282732 |
|  | 200 | 4.28 | [M + H]+ | 313.2747 | 18-oxononadecanoic acid | C_19_H_36_O_3_ | 312.266 | 4.39 | CID 5312917 |
|  | 299 | 4.92 | [M + H]+ | 267.2693 | Olealdehyde (a) | C_18_H_34_O | 266.261 | 3.72 | CID17029 |
|  | 300 | 4.67 | [M + H]+ | 267.2693 | Olealdehyde (b) | C_18_H_34_O | 266.261 | 3.72 | CID17029 |
|  | 329 | 0.93 | [M + H]+ | 202.1805 | 11-aminoundecanoic acid | C_11_H_23_NO_2_ | 201.173 | 1.09 | CID 17083 |
|  | 360 | 7.93 | [M + H]+ | 596.6016 | 2-[(2-Hydroxyethyl)(octadecyl)amino]ethyl hexadecanoate | C_38_H_77_NO_3_ | 595.59 | 7.25 | CID 71326110 |
| Indoles | 486 | 0.75 | [M + H]+ | 188.0708 | Indole-3-acrylic acid (a) | C_11_H_9_NO_2_ | 187.063 | 2.78 | CID 5375048 |
|  | 494 | 1.53 | [M + H]+ | 188.0708 | Indole-3-acrylic acid (b) | C_11_H_9_NO_2_ | 187.063 | 2.78 | CID 5375048 |
| Peptides | 18 | 2.52 | [M + NA]+ | 568.2972 | Pro-Gln-Arg-Phe amide trifluoroacetate salt | C_25_H_39_N_9_O_5_ | 545.307 | 1.80 | CID 3382578 |
|  | 47 | 2.45 | [M + K]+ | 257.0577 | L-Alanyl-D-glutamate | C_8_H_14_N_2_O_5_ | 218.0903 | 19.44 | C20957 |
| Organic acids | 285 | 2.04 | [M + H]+ | 191.0707 | 1-naphthoic acid | C_11_H_10_O_3_ | 190.063 | 2.21 | C15102 |
|  | 547 | 2.03 | [M + H]+ | 123.0441 | Benzoic acid (a) | C_7_H_6_O_2_ | 122.037 | -1.47 | C00180 |
|  | 580 | 1.84 | [M + H]+ | 123.0441 | Benzoic acid (b) | C_7_H_6_O_2_ | 122.037 | -1.47 | C00180 |
|  | 545 | 1.35 | [M + H]+ | 327.0687 | 5-Galloylshikimic acid (a) | C_14_H_14_O_9_ | 326.064 | -7.91 | CID 460897 |
|  | 585 | 0.7 | [M + H]+ | 327.0687 | 5-Galloylshikimic acid (b) | C_14_H_14_O_9_ | 326.064 | -7.91 | CID 460897 |
|  | 433 | 2.18 | [M + H]+ | 285.0612 | (2R,3R)-2-(2-Methoxybenzoyloxy)-3-hydroxysuccinic acid | C_12_H_12_O_8_ | 284.053 | 3.06 | CID 70014677 |
|  | 440 | 1.79 | [M + H]+ | 181.0863 | 3-(2-hydroxypropyl)benzoic acid | C_10_H_12_O_3_ | 180.079 | 0.28 | CID 67568076 |
|  | 97 | 7.76 | [M + H]+ | 593.4588 | 2-triacontanoylbenzenesulfonic acid | C_36_H_64_O_4_S | 592.453 | -2.50 | CID 23652140 |
| Inorganic acid | 44 | 12.83 | [M + H]+ | 258.917 | Triphosphoric Acid | H_5_O_10_P_3_ | 257.91 | -1.09 | C00536 |
| Trihydroxybenzoic acids | 462 | 1.78 | [M + H]+ | 315.0713 | Norbergenin | C_13_H_14_O_9_ | 314.064 | 0.06 | CID 73192 |
| Purine base | 467 | 1.33 | [M + H]+ | 268.1045 | Adenosine (a) | C_10_H_13_N_5_O_4_ | 267.097 | 0.82 | C00212 |
|  | 586 | 0.7 | [M + H]+ | 268.1045 | Adenosine (b) | C_10_H_13_N_5_O_4_ | 267.097 | 0.82 | C00212 |
|  | 141 | 0.68 | [M + H]+ | 136.0615 | Adenine (a) | C_5_H_5_N_5_ | 135.054 | 1.63 | C00147 |
|  | 310 | 1.33 | [M + H]+ | 136.0617 | Adenine (b) | C_5_H_5_N_5_ | 135.054 | 1.63 | C00147 |
| Monolignols | 309 | 1.76 | [M + NA]+ | 395.1316 | Syringin | C_17_H_24_O_9_ | 372.142 | 0.89 | C01533 |
|  | 372 | 1.78 | [M + NA]+ | 365.1212 | Coniferin | C_16_H_22_O_8_ | 342.1315 | 1.46 | C00761 |
| Phenolic acid | 367 | 2.9 | [M + H]+ | 280.2639 | Crucigasterin E | C_18_H_33_NO | 279.256 | 2.22 | CID 46937368 |
| Sugars | 495 | 1.78 | [M + NA]+ | 507.0752 | 4-O,6-O-Digalloyl-D-glucopyranose | C_20_H_20_O_14_ | 484.085 | 2.02 | CID 101630404 |
| Lumazine derivative | 100 | 1.51 | [M + K]+ | 369.0804 | reduced 6-(hydroxymethyl)-8-(1-D-ribityl)lumazine | C_20_H_16_O_7_ | 330.1176 | -1.03 | CHEBI:70986 |
| 2-benzofuran-1(3H)-one derivative | 268 | 3.42 | [M + H]+ | 205.086 | 3-Butylidene-7-hydroxyphthalide | C_12_H_12_O_3_ | 204.0786 | 0.34 | C09921 |
| Others | 3 | 2.9 | [M + H]+ | 415.212 | 2-[4-(4-methoxyphenyl)-3-methyl-pyrazolo[3,4-b]pyridin-1-yl]-N-(1-phenylpropyl)acetamide | C_25_H_26_N_4_O_2_ | 414.206 | -3.10 | CID 19506043 |
|  | 20 | 0.42 | [M + H]+ | 261.0582 | 8-methoxy-4,5-dihydrobenzo[g]benzothiophene-2-carboxylic acid | C_14_H_12_O_3_S | 260.051 | -0.35 | CID 81605969 |
|  | 29 | 0.41 | [M + H]+ | 203.0529 | N-(3-Nitramido-4,5-dihydropyridazin-4-yl)nitramide | C_4_H_6_N_6_O_4_ | 202.045 | 3.07 | CID 419333 |
|  | 34 | 8.09 | [M + H]+ | 753.4297 | 4-[[4-(benzenesulfonyl)-5-oxido-1,2,5-oxadiazol-5-ium-3-yl]oxy]butan-2-yl (4aS,6aR,6aS,6bR,8aR,10S,12aR,14bS)-10-hydroxy-2,2,6a,6b,9,9,12a-heptamethyl-1,3,4,5,6,6a,7,8,8a,10,11,12,13,14b-tetradecahydropicene-4a-carboxylate | C_42_H_60_N_2_O_8_S | 752.407 | 20.49 | CID 24949892 |
|  | 40 | 2.15 | [M + H]+ | 494.1245 | (2S)-2-amino-5-[[(1S)-2-(carboxymethylamino)-1-[(8-hydroxy-3-methyl-1,4-dioxo-2-naphthyl)sulfanylmethyl]-2-oxo-ethyl]amino]-5-oxo-pentanoic acid | C_21_H_23_N_3_O_9_S | 493.116 | 2.47 | CID 52942238 |
|  | 41 | 3.21 | [M + H]+ | 291.2536 | 5-isopropyl-N6-methyl-N4-[2-(4-methylcyclohexyl)ethyl]pyrimidine-4,6-diamine | C_17_H_30_N_4_ | 290.247 | -2.34 | CID 80822319 |
|  | 49 | 12.83 | [M + NA]+ | 356.8936 | 5-[(3,5-dichloro-4-pyridyl)sulfanyl]-4-nitro-thiophene-2-carbaldehyde | C_10_H_4_Cl_2_N_2_O_3_S_2_ | 333.904 | 1.14 | CID 87562543 |
|  | 50 | 2.44 | [M + H]+ | 359.1494 | 2-[2-[(3-cyano-2-pyridyl)oxy]anilino]-N-(p-tolyl)acetamide (a) | C_21_H_18_N_4_O_2_ | 358.143 | -2.46 | CID 60537784 |
|  | 51 | 3.24 | [M + NH4]+ | 335.2798 | N-[1-(3-Aminophenyl)ethylideneamino]undecanamide | C_19_H_31_N_3_O | 317.247 | -3.34 | CID 3616475 |
|  | 81 | 2.44 | [M + NA]+ | 381.1314 | 2-[2-[(3-cyano-2-pyridyl)oxy]anilino]-N-(p-tolyl)acetamide (b) | C_21_H_18_N_4_O_2_ | 358.143 | -2.29 | CID 60537784 |
|  | 102 | 0.42 | [M + H]+ | 247.0425 | 1-(3-hydroxy-2-thienyl)-3-phenyl-propane-1,3-dione | C_13_H_10_O_3_S | 246.035 | 0.89 | CID 13004301 |
|  | 121 | 0.44 | [M + H]+ | 229.0317 | 9-hydroxy-2-thioxo-3H-phenalen-1-one | C_13_H_8_O_2_S | 228.025 | -2.41 | CID 88740582 |
|  | 258 | 3.13 | [M + NA]+ | 391.1649 | Phthalic acid, 2-chloroethyl decyl ester | C_20_H_29_ClO_4_ | 368.175 | 1.85 | CID 6424038 |
|  | 290 | 3.05 | [M + NA]+ | 555.2936 | 2-(2,6-dihydroxyphenyl)-6-(1-hydroxy-3-oxo-octadecyl)-2-methyl-6H-furo[3,4-d][1,3]dioxol-4-one | C_30_H_44_O_8_ | 532.304 | 0.71 | CID 25069128 |
|  | 297 | 4.92 | [M + H]+ | 359.3165 | 18-(2,3-dihydroxypropoxy)octadecan-2-one (a) | C_21_H_42_O_4_ | 358.308 | 3.32 | CID 21475594 |
|  | 298 | 4.67 | [M + H]+ | 359.3165 | 18-(2,3-dihydroxypropoxy)octadecan-2-one (b) | C_21_H_42_O_4_ | 358.308 | 3.32 | CID 21475594 |
|  | 345 | 7.79 | [M + H]+ | 579.5385 | N''-[3-[3-[[4-(5-tert-butyl-2-methyl-phenyl)-1-[3-(5-tert-butyl-2-methyl-phenyl)propyl]butyl]amino]propylamino]propyl]propane-1,3-diamine | C_38_H_66_N_4_ | 578.529 | 3.84 | CID 90674863 |
|  | 347 | 8.03 | [M + H]+ | 647.5638 | 2-[2-[4-(4,6-dimethyldecan-3-yloxy)-3-methyl-5-pent-4-ynyloxolan-2-yl]oxy-3,4,6-trimethylcyclohexyl]oxy-6-ethyl-3,4,5-trimethyloxane | C_41_H_74_O_5_ | 646.554 | 3.90 | CID 90993583 |
|  | 350 | 7.79 | [M + H]+ | 619.5316 | 2-({16-[3-(Benzyloxy)-2-(octyloxy)propoxy]hexadecyl}oxy)tetrahydro-2H-pyran | C_39_H_70_O_5_ | 618.5223 | 3.27 | CSID:8992942 |
|  | 354 | 7.53 | [M + H]+ | 551.5068 | (Z)-19-methoxycarbonyltritriacont-9-enoic acid | C_35_H_66_O_4_ | 550.496 | 6.39 | CID 87313417 |
|  | 460 | 0.41 | [M + H]+ | 207.0357 | (2S)-2-(5-chloro-2-thienyl)-1-hydrazino-propan-2-ol | C_7_H_11_ClN_2_OS | 206.028 | 2.04 | CID 82077398 |
|  | 461 | 0.42 | [M + H]+ | 185.0534 | 1-azido-4-nitro-2H-triazin-5-amine | C_3_H_4_N_8_O_2_ | 184.046 | 0.65 | CID 22213878 |

**Table S2. Two-way ANOVA results for comparison of metabolites regulation between *Nepenthes* species (n = 12) and metabolite in response to the environmental condition.**

|  | **Sum of squares** | **df** | **Mean square** | **F** | **p (same)** |
| --- | --- | --- | --- | --- | --- |
| **Sample:** | 2054.4 | 11 | 186. 8 | 63.6 | 6.3E-135 |
| **metabolite:** | 41796.8 | 124 | 337. 1 | 114.8 | < 0.001 |
| **Interaction:** | 27132.6 | 1364 | 19. 9 | 6.8 | < 0.001 |
| **Within:** | 17615.6 | 6000 | 2.9 |  |  |
| **Total:** | 88599.4 | 7499 |  |  |  |

**Table S3. Tukey’s post-hoc results for comparison of each *Nepenthes* species under the provided environmental condition. HL – Highland condition; IL – Intermediate condition; LL – Lowland condition.**

|  | amp HL | amp IL | amp LL | min HL | min IL | min LL | north HL | north IL | north LL | raff HL | raff IL | raff LL |
| --- | --- | --- | --- | --- | --- | --- | --- | --- | --- | --- | --- | --- |
| amp HL |  | 0.4547 | 1.2E-12 | 1.32E-12 | 1.33E-12 | 1.31E-12 | 0.9368 | 0.7910 | 0.7212 | 0.7792 | 0.0638 | 0.0115 |
| amp IL | 0.4547 |  | 1.32E-12 | 1.33E-12 | 2.25E-10 | 2.43E-12 | 0.9997 | 1 | 1 | 0.0016 | 2.32E-06 | 1.1E-07 |
| amp LL | 1.2E-12 | 1.32E-12 |  | 0.9579 | 0.1780 | 0.6256 | 1.32E-12 | 1.32E-12 | 1.32E-12 | 1.2E-12 | 1.2E-12 | 1.2E-12 |
| min HL | 1.32E-12 | 1.33E-12 | 0.9579 |  | 0.9682 | 1 | 1.34E-12 | 1.29E-12 | 1.3E-12 | 1.2E-12 | 1.2E-12 | 1.2E-12 |
| min IL | 1.33E-12 | 2.25E-10 | 0.1780 | 0.9682 |  | 0.9999 | 1.79E-12 | 7.88E-12 | 1.65E-11 | 1.24E-12 | 1.2E-12 | 1.2E-12 |
| min LL | 1.31E-12 | 2.43E-12 | 0.6256 | 1 | 0.9999 |  | 1.32E-12 | 1.36E-12 | 1.41E-12 | 1.2E-12 | 1.2E-12 | 1.2E-12 |
| north HL | 0.9368 | 0.9997 | 1.32E-12 | 1.34E-12 | 1.79E-12 | 1.32E-12 |  | 1 | 1 | 0.03492 | 0.0002 | 1.2E-05 |
| north IL | 0.7910 | 1 | 1.32E-12 | 1.29E-12 | 7.88E-12 | 1.36E-12 | 1 |  | 1 | 0.01104 | 3.13E-05 | 1.91E-06 |
| north LL | 0.7212 | 1 | 1.32E-12 | 1.3E-12 | 1.65E-11 | 1.41E-12 | 1 | 1 |  | 0.00728 | 1.76E-05 | 1.01E-06 |
| raff HL | 0.7792 | 0.0016 | 1.2E-12 | 1.2E-12 | 1.24E-12 | 1.2E-12 | 0.03492 | 0.01104 | 0.00728 |  | 0.9766 | 0.7973 |
| raff IL | 0.0638 | 2.32E-06 | 1.2E-12 | 1.2E-12 | 1.2E-12 | 1.2E-12 | 0.0002 | 3.13E-05 | 1.76E-05 | 0.9766 |  | 1 |
| raff LL | 0.0115 | 1.1E-07 | 1.2E-12 | 1.2E-12 | 1.2E-12 | 1.2E-12 | 1.2E-05 | 1.91E-06 | 1.01E-06 | 0.7973 | 1 |  |

**Table S4.** Similarly, and individually expressed thermal stressed metabolites among the 4 *Nepenthes* species in response to the provided environmental conditions.

| 12 common elements in "minima", "ampullaria", "northiana" and "rafflesiana" | (H) Epiafzelechin 3-O-gallate-(4beta->6)-epigallocatechin 3-O-gallate  (H) 4-[[4-(benzenesulfonyl)-5-oxido-1,2,5-oxadiazol-5-ium-3-yl]oxy]butan-2-yl (4aS,6aR,6aS,6bR,8aR,10S,12aR,14bS)-10-hydroxy-2,2,6a,6b,9,9,12a-heptamethyl-1,3,4,5,6,6a,7,8,8a,10,11,12,13,14b-tetradecahydropicene-4a-carboxylate  (H) Berberastine  (H) 1-naphthoic acid  (H) adenine (a)  (H) adenine (b)  (L) L-Tryptophan  (L) 18-oxononadecanoic acid  (L) Olealdehyde (a)  (L) Olealdehyde (b)  (L) Indole-3-acrylic acid (a)  (L) 2-[2-[(3-cyano-2-pyridyl)oxy]anilino]-N-(p-tolyl)acetamide (a) |
| --- | --- |
| 8 common elements in "minima", "ampullaria" and "northiana" | (L) Anatoxin a(s)  (L) 2-[(2-Hydroxyethyl)(octadecyl)amino]ethyl hexadecanoate  (L) 2-[2-[(3-cyano-2-pyridyl)oxy]anilino]-N-(p-tolyl)acetamide (b)  (L) 18-(2,3-dihydroxypropoxy)octadecan-2-one (b)  (L) N''-[3-[3-[[4-(5-tert-butyl-2-methyl-phenyl)-1-[3-(5-tert-butyl-2-methyl-phenyl)propyl]butyl]amino]propylamino]propyl]propane-1,3-diamine  (L) 2-[2-[4-(4,6-dimethyldecan-3-yloxy)-3-methyl-5-pent-4-ynyloxolan-2-yl]oxy-3,4,6-trimethylcyclohexyl]oxy-6-ethyl-3,4,5-trimethyloxane  (L) 2-({16-[3-(Benzyloxy)-2-(octyloxy)propoxy]hexadecyl}oxy)tetrahydro-2H-pyran  (L) (Z)-19-methoxycarbonyltritriacont-9-enoic acid |
| 7 common elements in "ampullaria", "northiana" and "rafflesiana" | (H) Kaempferol 3-O-beta-D-xyloside(b)  (H) Pro-Gln-Arg-Phe amide trifluoroacetate salt  (H) Adenosine (a)  (H) Adenosine (b)  (H) (2S)-2-amino-5-[[(1S)-2-(carboxymethylamino)-1-[(8-hydroxy-3-methyl-1,4-dioxo-2-naphthyl)sulfanylmethyl]-2-oxo-ethyl]amino]-5-oxo-pentanoic acid  (H) 5-isopropyl-N6-methyl-N4-[2-(4-methylcyclohexyl)ethyl]pyrimidine-4,6-diamine  (H) Phthalic_acid,2-chloroethyl-decyl-ester |
| 5 common elements in "minima", "ampullaria" and "rafflesiana" | (H) 5-Deoxyleucocyanidin  (L) Kaempferol 3-O-beta-D-xyloside(a)  (L) Indole-3-acrylic acid (b)  (L) 5-Galloylshikimic acid (b)  (H) Triphosphoric_Acid |
| 2 common elements in "minima", "northiana" and "rafflesiana" | (L) Kaempferol 3-O-glucoside  (L) (2S)-2-(3,4-dihydroxyphenyl)-8-[(2S,4R)-2-(3,4-dihydroxyphenyl)-7-hydroxy-5-[(2S,3R,4S,5S,6R)-3,4,5-trihydroxy-6-(hydroxymethyl)oxan-2-yl]oxy-3,4-dihydro-2H-chromen-4-yl]-5,7-dihydroxy-2,3-dihydrochromen-4-one |
| 6 common elements in "ampullaria" and "northiana" | (H) Baicalein (b)  (L) Baicalein (c)  (L) 2-[4-(4-methoxyphenyl)-3-methyl-pyrazolo[3,4-b]pyridin-1-yl]-N-(1-phenylpropyl)acetamide  (L) N-(3-Nitramido-4,5-dihydropyridazin-4-yl)nitramide  (L) 1-(3-hydroxy-2-thienyl)-3-phenyl-propane-1,3-dione  (L) 2-(2,6-dihydroxyphenyl)-6-(1-hydroxy-3-oxo-octadecyl)-2-methyl-6H-furo[3,4-d][1,3]dioxol-4-one |
| 7 common elements in "ampullaria" and "rafflesiana" | (H) Rutin  (H) L-Alanyl-D-glutamate  (H) 3-(2-hydroxypropyl)benzoic acid  (H) Norbergenin ([antioxidant activities) possible protect the plant from cool induced ros?](http://herbapolonica.pl/magazines-files/3110328-Rastogi & Rawat.pdf) And ………….[DPPH positive.](https://www.sciencedirect.com/science/article/abs/pii/S1874390009000597)  (H) 3-Butylidene-7-hydroxyphthalide  (H) 5-[(3,5-dichloro-4-pyridyl)sulfanyl]-4-nitro-thiophene-2-carbaldehyde  (H) N-[1-(3-Aminophenyl)ethylideneamino]undecanamide |
| 8 common elements in "minima" and "rafflesiana" | (H) Quercetin 3-(2''-galloyl-alpha-L-arabinopyranoside) (a)  (H) Baicalin  (L) Baicalein (b)  (H) Luteolin  (L) myrciacitrin I  (L) L-Arginine  (H) 2-(2,6-dihydroxyphenyl)-6-(1-hydroxy-3-oxo-octadecyl)-2-methyl-6H-furo[3,4-d][1,3]dioxol-4-one  (L) (2S)-2-(5-chloro-2-thienyl)-1-hydrazino-propan-2-ol |
| 4 common elements in "minima" and "northiana" | (H) Quercetin 3-(2''-galloyl-alpha-L-arabinopyranoside) (b)  (L) 4-O,6-O-Digalloyl-D-glucopyranose  (L) 9-hydroxy-2-thioxo-3H-phenalen-1-one  (L) 8-methoxy-4,5-dihydrobenzo[g]benzothiophene-2-carboxylic acid |
| 9 common elements in "minima" and "ampullaria" | (H) 4,2'-Dihydroxy-4',6'-dimethoxychalcone 4-glucoside  (H) Peruvianoside II  (L) Baicalein (a)  (L) Norvaline  (L) 11-aminoundecanoic acid  (H) 3,5,7-trihydroxy-2-[3,4,5-trihydroxy-2-[2,3,4-trihydroxy-6-(3,5,7-trihydroxychroman-2-yl)phenyl]phenyl]chromen-4-one (b)  (H) Benzoic acid (a)  (H) Benzoic acid (b)  (L) 18-(2,3-dihydroxypropoxy)octadecan-2-one (a) |
| 8 common elements in "northiana" and "rafflesiana" | (H) minaprine  (H) Baicalein (a)  (L) Butin (b)  (H) 7-Dodecenoic acid  (H) 11-aminoundecanoic acid  (L) Benzoic acid (b)  (L) Syringin  (H) Crucigasterin E |
| 41 elements included exclusively in "minima" | (H) Catechin 5-O-gallate  (H) Baicalein (c)  (H) Afzelin  (H) Butin (a)  (H) Butin (b)  (H) (2R,3R)-2-(2-Methoxybenzoyloxy)-3-hydroxysuccinic acid  (H) N-(3-Nitramido-4,5-dihydropyridazin-4-yl)nitramide  (L) Kaempferol 3-O-beta-D-xyloside(b)  (L) 2-triacontanoylbenzenesulfonic acid  (L) Trigonelline  (L) Isoleucine  (L) (2S)-2-amino-5-[[(1S)-2-(carboxymethylamino)-1-[(8-hydroxy-3-methyl-1,4-dioxo-2-naphthyl)sulfanylmethyl]-2-oxo-ethyl]amino]-5-oxo-pentanoic acid  1 Quercetin-3-O-rhamnoside  1 Quercetin 3-(6''-galloylglucoside)  1 minaprine  1 Quercetin  1 Rutin  1 6,8-Dihydroxykaempferol 3-rutinoside  1 miquelianin (a)  1 miquelianin (b)  1 7-Dodecenoic acid  1 Pro-Gln-Arg-Phe amide trifluoroacetate salt  1 L-Alanyl-D-glutamate  1 5-Galloylshikimic acid (a)  1 3-(2-hydroxypropyl)benzoic acid  1 Norbergenin  1 Adenosine (a)  1 Adenosine (b)  1 Syringin  1 Coniferin  1 Crucigasterin E  1 reduced 6-(hydroxymethyl)-8-(1-D-ribityl)lumazine  1 3-Butylidene-7-hydroxyphthalide  1 2-[4-(4-methoxyphenyl)-3-methyl-pyrazolo[3,4-b]pyridin-1-yl]-N-(1-phenylpropyl)acetamide  1 5-isopropyl-N6-methyl-N4-[2-(4-methylcyclohexyl)ethyl]pyrimidine-4,6-diamine  1 5-[(3,5-dichloro-4-pyridyl)sulfanyl]-4-nitro-thiophene-2-carbaldehyde  1 N-[1-(3-Aminophenyl)ethylideneamino]undecanamide  1 1-(3-hydroxy-2-thienyl)-3-phenyl-propane-1,3-dione  1 Phthalic_acid,2-chloroethyl-decyl-ester  1 3,5,7-trihydroxy-2-[3,4,5-trihydroxy-2-[2,3,4-trihydroxy-6-(3,5,7-trihydroxychroman-2-yl)phenyl]phenyl]chromen-4-one (a)  1 1-azido-4-nitro-2H-triazin-5-amine |
| 35 elements included exclusively in "ampullaria" | (H) 9-hydroxy-2-thioxo-3H-phenalen-1-one  (H) (2S)-2-(5-chloro-2-thienyl)-1-hydrazino-propan-2-ol  (H) 8-methoxy-4,5-dihydrobenzo[g]benzothiophene-2-carboxylic acid  (H) Trigonelline  (H) Isoleucine  (H) Coniferin  (L) Crucigasterin E  (L) reduced 6-(hydroxymethyl)-8-(1-D-ribityl)lumazine  (L) 7-Dodecenoic acid  (L) minaprine  2 Quercetin-3-O-rhamnoside  2 Kaempferol 3-O-glucoside  2 Quercetin 3-(6''-galloylglucoside)  2 Catechin 5-O-gallate  2 Quercetin  2 Quercetin 3-(2''-galloyl-alpha-L-arabinopyranoside) (a)  2 Quercetin 3-(2''-galloyl-alpha-L-arabinopyranoside) (b)  2 Baicalin  2 6,8-Dihydroxykaempferol 3-rutinoside  2 Afzelin  2 miquelianin (a)  2 miquelianin (b)  2 Luteolin  2 Butin (a)  2 Butin (b)  2 myrciacitrin I  2 (2S)-2-(3,4-dihydroxyphenyl)-8-[(2S,4R)-2-(3,4-dihydroxyphenyl)-7-hydroxy-5-[(2S,3R,4S,5S,6R)-3,4,5-trihydroxy-6-(hydroxymethyl)oxan-2-yl]oxy-3,4-dihydro-2H-chromen-4-yl]-5,7-dihydroxy-2,3-dihydrochromen-4-one  2 L-Arginine  2 5-Galloylshikimic acid (a)  2 (2R,3R)-2-(2-Methoxybenzoyloxy)-3-hydroxysuccinic acid  2 3,5,7-trihydroxy-2-[3,4,5-trihydroxy-2-[2,3,4-trihydroxy-6-(3,5,7-trihydroxychroman-2-yl)phenyl]phenyl]chromen-4-one (a)  2 2-triacontanoylbenzenesulfonic acid  2 Syringin  2 4-O,6-O-Digalloyl-D-glucopyranose  2 1-azido-4-nitro-2H-triazin-5-amine |
| 42 elements included exclusively in "northiana" | (H) 5-Galloylshikimic acid (a)  (H) Kaempferol 3-O-beta-D-xyloside(a)  (H) myrciacitrin I  (H) Norvaline  (L) Rutin  (L) 6,8-Dihydroxykaempferol 3-rutinoside  (L) Butin (a)  (L) Benzoic acid (a)  (L) Quercetin 3-(6''-galloylglucoside)  (L) Quercetin  (L) (2R,3R)-2-(2-Methoxybenzoyloxy)-3-hydroxysuccinic acid  (L) 3-(2-hydroxypropyl)benzoic acid  (L) Triphosphoric_Acid  (L) Norbergenin  (L) 5-[(3,5-dichloro-4-pyridyl)sulfanyl]-4-nitro-thiophene-2-carbaldehyde  (L) N-[1-(3-Aminophenyl)ethylideneamino]undecanamide  3 Quercetin-3-O-rhamnoside  3 5-Deoxyleucocyanidin  3 Catechin 5-O-gallate  3 4,2'-Dihydroxy-4',6'-dimethoxychalcone 4-glucoside  3 Quercetin 3-(2''-galloyl-alpha-L-arabinopyranoside) (a)  3 Peruvianoside II  3 Baicalin  3 Afzelin  3 miquelianin (a)  3 miquelianin (b)  3 Luteolin  3 3,5,7-trihydroxy-2-[3,4,5-trihydroxy-2-[2,3,4-trihydroxy-6-(3,5,7-trihydroxychroman-2-yl)phenyl]phenyl]chromen-4-one  3 3,5,7-trihydroxy-2-[3,4,5-trihydroxy-2-[2,3,4-trihydroxy-6-(3,5,7-trihydroxychroman-2-yl)phenyl]phenyl]chromen-4-one (a)  3 Trigonelline  3 L-Arginine  3 Isoleucine  3 Indole-3-acrylic acid (b)  3 L-Alanyl-D-glutamate  3 5-Galloylshikimic acid (b)  3 2-triacontanoylbenzenesulfonic acid  3 Coniferin  3 reduced 6-(hydroxymethyl)-8-(1-D-ribityl)lumazine  3 3-Butylidene-7-hydroxyphthalide  3 18-(2,3-dihydroxypropoxy)octadecan-2-one (a)  3 (2S)-2-(5-chloro-2-thienyl)-1-hydrazino-propan-2-ol  3 1-azido-4-nitro-2H-triazin-5-amine |
| 40 elements included exclusively in "rafflesiana" | (H) Quercetin 3-(6''-galloylglucoside)  (H) 2-[(2-Hydroxyethyl)(octadecyl)amino]ethyl hexadecanoate  (H) 18-(2,3-dihydroxypropoxy)octadecan-2-one (a)  (H) N''-[3-[3-[[4-(5-tert-butyl-2-methyl-phenyl)-1-[3-(5-tert-butyl-2-methyl-phenyl)propyl]butyl]amino]propylamino]propyl]propane-1,3-diamine  (H) 2-[2-[4-(4,6-dimethyldecan-3-yloxy)-3-methyl-5-pent-4-ynyloxolan-2-yl]oxy-3,4,6-trimethylcyclohexyl]oxy-6-ethyl-3,4,5-trimethyloxane  (H) 2-({16-[3-(Benzyloxy)-2-(octyloxy)propoxy]hexadecyl}oxy)tetrahydro-2H-pyran  (H) (Z)-19-methoxycarbonyltritriacont-9-enoic acid  (H) 2-triacontanoylbenzenesulfonic acid  (H) 4-O,6-O-Digalloyl-D-glucopyranose  (H) reduced 6-(hydroxymethyl)-8-(1-D-ribityl)lumazine  (H) 2-[4-(4-methoxyphenyl)-3-methyl-pyrazolo[3,4-b]pyridin-1-yl]-N-(1-phenylpropyl)acetamide  (L) 1-azido-4-nitro-2H-triazin-5-amine  (L) 3,5,7-trihydroxy-2-[3,4,5-trihydroxy-2-[2,3,4-trihydroxy-6-(3,5,7-trihydroxychroman-2-yl)phenyl]phenyl]chromen-4-one (a)  (L) 5-Galloylshikimic acid (a)  (L) miquelianin (a)  (L) miquelianin (b)  (L) 4,2'-Dihydroxy-4',6'-dimethoxychalcone 4-glucoside  (L) Quercetin 3-(2''-galloyl-alpha-L-arabinopyranoside) (b)  (L) Peruvianoside II  4 Quercetin-3-O-rhamnoside  4 Catechin 5-O-gallate  4 Quercetin  4 Baicalein (c)  4 6,8-Dihydroxykaempferol 3-rutinoside  4 Afzelin  4 Butin (a)  4 Trigonelline  4 Anatoxin a(s)  4 Isoleucine  4 Norvaline  4 Benzoic acid (a)  4 (2R,3R)-2-(2-Methoxybenzoyloxy)-3-hydroxysuccinic acid  4 Coniferin  4 N-(3-Nitramido-4,5-dihydropyridazin-4-yl)nitramide  4 2-[2-[(3-cyano-2-pyridyl)oxy]anilino]-N-(p-tolyl)acetamide (b)  4 1-(3-hydroxy-2-thienyl)-3-phenyl-propane-1,3-dione  4 9-hydroxy-2-thioxo-3H-phenalen-1-one  4 18-(2,3-dihydroxypropoxy)octadecan-2-one (b)  4 3,5,7-trihydroxy-2-[3,4,5-trihydroxy-2-[2,3,4-trihydroxy-6-(3,5,7-trihydroxychroman-2-yl)phenyl]phenyl]chromen-4-one (b)  4 8-methoxy-4,5-dihydrobenzo[g]benzothiophene-2-carboxylic acid |
